# Pirin Transcriptionally Regulates PLA2G4A To Inhibit Ferroptosis in Colorectal Cancer via Lipid Profile Remodeling

**DOI:** 10.1002/advs.202516385

**Published:** 2025-12-16

**Authors:** Wei Shi, Yue Qi Ong, Prativa Majee, Esther SM Wong, Amhed Missael Vargas Velazquez, Kerem Fidan, Pierce Kah‐Hoe Chow, Wai Leong Tam, Ker Kan Tan, Iain Beehuat Tan, Vinay Tergaonkar

**Affiliations:** ^1^ Laboratory of NFκB Signaling Institute of Molecular and Cell Biology (IMCB) Agency for Science Technology and Research (A*STAR) 61 Biopolis Drive Proteos Singapore 138673 Singapore; ^2^ Programme in Translational and Clinical Liver Research National Cancer Centre Singapore Singapore 169610 Singapore; ^3^ Genome Institute of Singapore Agency for Science Technology and Research (A*STAR) 60 Biopolis Street Genome Singapore 138672 Singapore; ^4^ Department of Surgery Yong Loo Lin School of Medicine National University of Singapore Level 8. 1E Kent Ridge Road Singapore 119228 Singapore; ^5^ Division of Medical Oncology National Cancer Centre Singapore 30 Hospital Boulevard Singapore 168583 Singapore

**Keywords:** colorectal cancer, ferroptosis, lipid metabolism, Pirin, PLA2G4A

## Abstract

Ferroptosis, an iron‐dependent cell death driven by lipid peroxidation, is a promising therapeutic target in colorectal cancer (CRC); however, its regulation remains unclear. Here, Pirin (PIR) is recognized as a ferroptosis suppressor that acts through lipid remodeling. PIR is upregulated in CRC tissues, correlating with reduced ferroptosis sensitivity and enhanced tumor growth, whereas PIR loss restricts CRC progression in vivo and in vitro. Intestinal epithelium–specific *PIR* deletion limits AOM/DSS‐induced tumorigenesis by increasing lipid peroxidation and promoting ferroptosis. Mechanistically, ferroptosis triggers a compensatory NRF2‐PIR axis, in which NRF2 binds to the PIR promoter to induce its expression. PIR deficiency downregulates PLA2G4A (encoding cPLA2α), a key arachidonic‐acid–metabolizing enzyme involved in ferroptosis control. Lipidomics has shown that PIR loss increases polyunsaturated fatty acid (PUFA)‐containing phospholipids and decreases monounsaturated (MUFA) and saturated (SFA) species, shifting membranes toward a ferroptosis‐permissive state. Restoration of PLA2G4A rescues ferroptosis resistance in PIR‐deficient cells. Targeting this pathway, either by pharmacologic inhibition of PLA2G4A with AACOCF3 or by genetic disruption of the PIR–PLA2G4A axis, enhances the efficacy of ferroptosis inducers and suppresses CRC progression. This study defines an NRF2–PIR–PLA2G4A circuit that governs ferroptosis susceptibility via lipidome remodeling and highlights its therapeutic potential in CRC.

## Graphical abstract

This study identifies Pirin (PIR), an iron‐binding protein, as a critical ferroptosis suppressor in colorectal cancer through lipid membrane remodeling. PIR, induced by NRF2 during ferroptotic stress, transcriptionally regulates PLA2G4A to shift cellular lipid composition away from ferroptosis‐permissive polyunsaturated phospholipids. Disrupting the NRF2–PIR–PLA2G4A axis sensitizes tumors to ferroptosis inducers and restricts cancer progression, revealing a therapeutic vulnerability in colorectal cancer treatment.

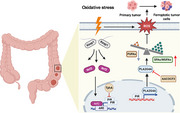

## 1 Introduction

Colorectal cancer (CRC) is the third most commonly diagnosed cancer and a major clinical challenge, especially in advanced stages, with a 5‐year survival rate of ≈12%.^[^ ^1^, ^2^ ^]^ Conventional interventions, including surgery, chemotherapy, targeted therapy, and immunotherapy, are often hindered by recurrence, drug resistance, and toxicity.^[^ ^3^ ^]^ These challenges highlight the need for novel biomarkers and molecular‐targeted therapies. In this context, recent research has shifted toward the development of pathway‐specific therapeutics, especially those targeting regulated cell death (RCD) pathways, which have emerged as promising strategies in the treatment of CRC. However, the molecular mechanisms underlying CRC pathogenesis, metastasis, and therapeutic resistance remain unclear. This highlights the urgent need to identify new biomarkers, clarify their functional roles, and develop targeted therapies. Considering the evasion of cell death is a hallmark of cancer,^[^ ^4^ ^]^ exploiting cell death pathways therapeutically remains an attractive strategy. However, apoptotic inducers such as Venetoclax^[^ ^5^ ^]^ have only recently demonstrated tangible clinical efficacy. This limited efficacy has generated increased interest in alternative cell death mechanisms,^[^ ^6^ ^]^ as they may have greater therapeutic potential and overcome resistance to conventional apoptosis‐targeted therapies in CRC.

Ferroptosis is a regulated, iron‐dependent, programmed cell death that is mechanistically distinct from apoptosis and necrosis.^[^ ^7^, ^8^ ^]^ Under normal conditions, it contributes to cellular homeostasis, but when dysregulated, it can drive a range of pathological processes.^[^ ^9^ ^]^ Ferroptosis plays a dual role in cancer: its suppression supports tumor growth and survival, whereas its activation represents a promising therapeutic strategy for multiple malignancies, including CRC.^[^ ^10^ ^]^ Canonical ferroptosis inducers (FINs), including erastin and RSL3, function by obstructing system x_c_^−^ or inhibiting glutathione peroxidase 4 (GPX4), respectively, thereby limiting cysteine uptake, depleting glutathione (GSH) biosynthesis, and disrupting regulatory processes associated with iron homeostasis and ferroptosis suppressor protein 1 (FSP1).^[^ ^11^ ^]^ Conversely, lipophilic antioxidants, iron chelators, lipid peroxidation inhibitors, and decreases in PUFA‐phospholipids all impede ferroptosis.^[^ ^12^ ^]^ Malignant cells evade ferroptotic death by activating or reprogramming adaptive stress response pathways, hence diminishing the effectiveness of the inducers. Ferroptosis has been established as a significant tumor‐suppressive mechanism,^[^ ^10^, ^12^ ^]^ and its activation enhances the therapeutic efficacy of immunotherapy,^[^ ^13^ ^]^ radiotherapy,^[^ ^14^ ^]^ and chemotherapy.^[^ ^15^ ^]^ Due to the genetic heterogeneity of cancer, we must elucidate the comprehensive regulation of ferroptosis within specific genetic contexts to develop targeted therapies. CRC shows a distinctive susceptibility to ferroptosis due to chronic inflammation and dietary factors that increase labile iron levels and lipid peroxidation substrates. Leveraging ferroptosis‐based approaches with standard therapies can enhance clinical outcomes. Consequently, a deeper understanding of ferroptosis regulation in CRC and how these pathways can be therapeutically leveraged remains a critical research priority.

Pirin (PIR), an iron‐binding protein that belongs to the cupin superfamily, has a two‐barrel domain containing non‐heme Fe (II) at its N‐terminus.^[^ ^16^ ^]^ It serves as a transcriptional coactivator for NFκB^[^ ^17^ ^]^ and exhibits quercetinase activity, contributing to redox modulation, kinase inhibition, and transcriptional regulation.^[^ ^18^ ^]^ PIR dysregulation has been implicated in multiple malignancies, including colorectal,^[^ ^19^ ^]^ breast,^[^ ^20^ ^]^ and prostate cancers,^[^ ^21^ ^]^ as well as melanoma.^[^ ^22^ ^]^ Nuclear factor erythroid 2‐related factor 2 (NRF2; encoded by NFE2L2) is the master regulator of antioxidant responses and a key modulator of PIR expression.^[^ ^19^ ^]^ NRF2 governs the transcription of oxidative stress response genes, including ferroptosis‐associated factors, such as glutathione synthesis enzymes and lipid peroxidation regulators. This suggests that PIR may modulate ferroptosis, although the mechanistic relationship between PIR and ferroptotic pathways in CRC remains poorly characterized.

The phospholipase A2 (PLA2) superfamily comprises more than 50 isoforms that hydrolyze fatty acids at the sn‐2 position to release arachidonic acid (AA).^[^ ^23^ ^]^ These enzymes are classified as secreted (sPLA2), cytosolic (cPLA2), calcium‐independent (iPLA2), and lipoprotein‐associated (Lp‐PLA2), each with distinct structural and regulatory characteristics.^[^ ^24^ ^]^ Several PLA2 isoforms have been implicated in the regulation of ferroptosis. Notably, iPLA2β (PLA2G6) attenuates ferroptosis,^[^ ^25^ ^]^ while Lp‐PLA2 inhibition by darapladib synergizes with GPX4 suppression to enhance ferroptotic cell death.^[^ ^26^ ^]^ In contrast, cPLA2, and especially PLA2G4A (cPLA2α), enhances the release of arachidonic acid (AA), increases lipid peroxidation, and promotes prostaglandin biosynthesis, thereby conferring context‐dependent pro‐ or anti‐ferroptotic effects.^[^ ^27^, ^28^ ^]^ PLA2G4A is also involved in inflammatory signaling and intestinal tumorigenesis,^[^ ^29^ ^]^ and is regulated by MAPK‐dependent phosphorylation.^[^ ^30^ ^]^ Although PLA2G4A overexpression is associated with chemotherapy resistance in gastric cancer,^[^ ^31^ ^]^ the therapeutic effect of cPLA2 inhibition appears to vary depending on the tumor type.

In this study, we show that PIR regulates ferroptosis, reshaping lipid profiles in CRC cells. We also show that ferroptosis induces the activation of the compensatory NRF2‐PIR signaling pathway, upregulating PIR to establish adaptive resistance. Mechanistically, PIR regulates PLA2G4A transcription, altering the balance between proferroptotic PUFA‐phospholipids and antiferroptotic MUFA/SFA‐phospholipids. Furthermore, we demonstrate that targeting the PIR‐PLA2G4A axis (genetic or pharmacological disruption) significantly sensitizes CRC cells to ferroptosis inducers, thereby inhibiting tumor progression in vitro and in vivo. These findings establish a key regulatory mechanism of the NRF2‐PIR‐PLA2G4A pathway in ferroptosis and provide a novel therapeutic strategy for enhancing ferroptosis‐based CRC treatment.

## 2 Results

### 2.1 PIR is Upregulated in CRC and Correlates with Ferroptosis Resistance

Given CRC's established link to iron metabolism and ferroptosis, there is compelling therapeutic evidence that targeting ferroptosis could lead to an effective therapeutic strategy.

**Figure 1:**
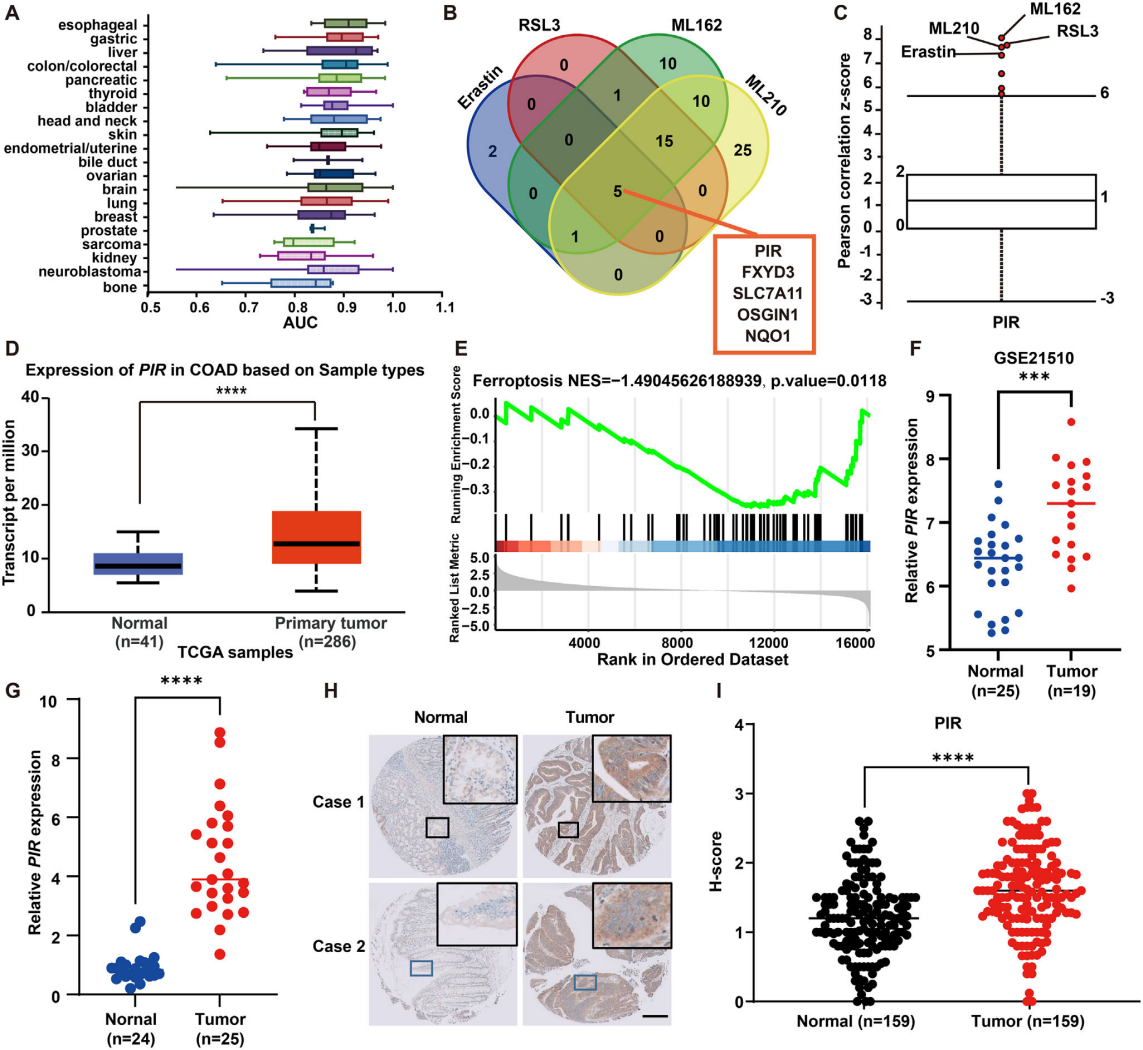
Pirin overexpression in CRC correlates with ferroptosis resistance. A) Erastin sensitivity across 20 cancer types via DepMap, quantified by AUC values (higher AUC indicates resistance). B) Venn diagrams identifying genes (z‐score > 7) with expression negatively correlating with ferroptosis inducer sensitivity (erastin, RSL3, ML162, ML210) in the CTRP dataset; PIR represents a key overlapping candidate. C) Correlation analysis between PIR expression and ferroptosis inducer resistance using CTRP. D) *PIR* transcriptional levels in colonic adenocarcinoma via the UALCAN portal. E) GSEA showing inverse correlation between PIR expression and ferroptosis pathway in TCGA‐COAD READ cohort. F) PIR transcriptional profiles in CRC specimens from the GEO repository (GSE21510) (normal, n = 25; tumor, n = 19). G) qRT‐PCR validation of *PIR* expression in paired CRC tissues (Normal, n = 24; Tumor, n = 25). H) PIR protein expression in CRC tissue microarray by immunohistochemistry. Representative images show differential PIR immunoreactivity between normal epithelium (left) and carcinoma (right). Scale bar: 200 µm. I) TMA‐based H‐score quantification confirming elevated PIR protein in CRC specimens (Normal, n = 159; Tumor, n = 159). Data are presented as mean ± SD, with n = 44 (F), 25 (G), or 159 (I) independent repeats. ^***^ *P* < 0.001, ^****^ *P* < 0.0001 by two‐tailed unpaired Student's *t*‐test (D, F, G, and I).

The DepMap database was used to investigate the sensitivity of 20 tumor types to erastin, a prototypical ferroptosis inducer, and the results showed that CRC was the fourth least sensitive tumor type (**Figure** **1**A). This indicates that resistance to ferroptosis is a critical mechanism in CRC tumorigenesis. To elucidate the mechanism of ferroptosis resistance, the correlation between gene expression and resistance to four ferroptosis inducers (FINs), namely erastin, RSL3, ML162, and ML210, across 654 cancer cell lines, was analyzed using the Cancer Therapy Response Portal (CTRP).^[^ ^32^ ^]^ PIR was identified among five common biomarkers that were correlated with resistance to FINs (Pearson correlation z‐score > 7; Figure 1B,C). Elevated PIR expression has been predicted in multiple CRC datasets. TCGA‐COAD analysis using the UALCAN database demonstrated much higher PIR expression in colon adenocarcinoma tissues than in normal tissues (Figure 1D). Gene Set Enrichment Analysis (GSEA) of the TCGA dataset revealed a significant negative association between PIR expression and ferroptosis in neoplastic colon adenocarcinoma samples (NES = −1.49, adjusted *P* = 0.0118, Figure 1E). Similarly, the GEO dataset GSE21510 revealed elevated PIR expression in CRC tissues (Figure 1F). qPCR analysis of 25 paired CRC samples (Figure 1G) and immunohistochemical staining of tissue microarrays from 159 patients with CRC (Figure 1H,I) confirmed significant PIR upregulation in CRC compared with that in adjacent normal tissues (Figure 1G–I). As indicated in our initial screening, PIR's iron‐binding properties, its association with CRC, and its regulation of ferroptosis (Figure 1B), motivated us to investigate its mechanistic role as a signaling node linking ferroptosis and CRC pathogenesis.

### 2.2 PIR Deficiency Promotes Ferroptotic CRC Cell Death In Vitro

**Figure 2:**
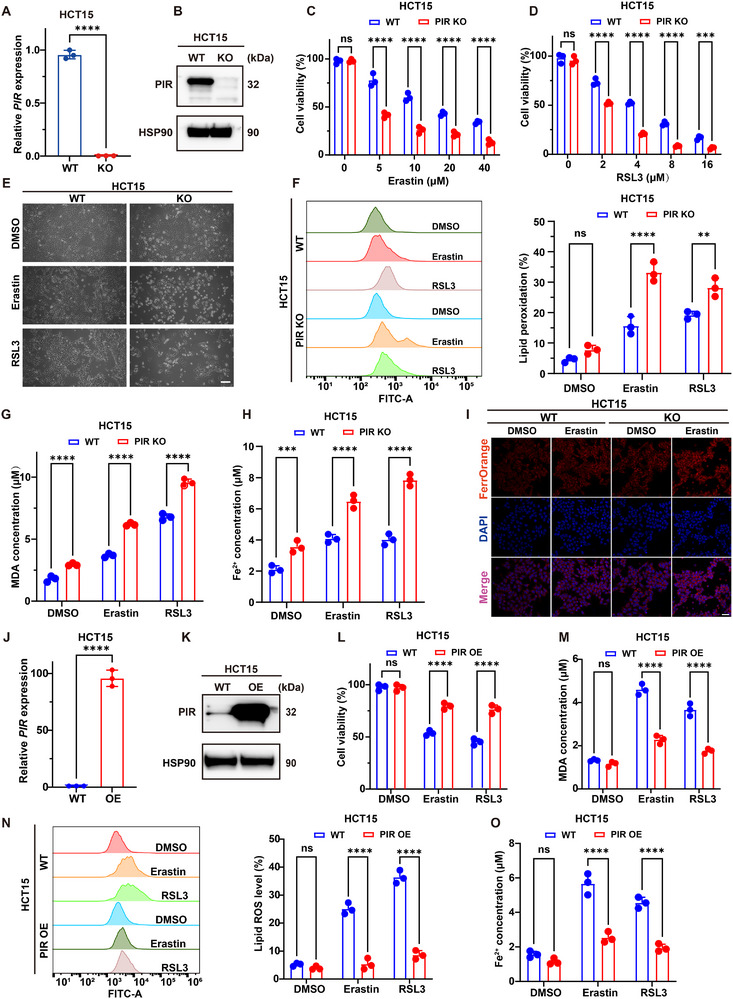
PIR modulates ferroptosis susceptibility in CRC cells. A) qRT‐PCR analysis of *PIR* mRNA in wild‐type (WT) and PIR‐knockout (KO) HCT15 cells. B) Western blot confirming PIR protein ablation in KO cells. C, D) Dose‐dependent viability reduction in PIR‐KO versus WT HCT15 cells treated with erastin or RSL3 (24 h). E) Phase‐contrast microscopy showing ferroptosis‐associated morphological changes in erastin/RSL3‐treated PIR‐depleted cells. Scale bar: 100 µm. F) Flow cytometric quantification of C11‐BODIPY 581/591 fluorescence showing elevated lipid peroxidation in PIR‐KO cells post‐ferroptosis induction. G) Intracellular MDA levels in HCT15 WT and PIR‐KO cells following erastin (10 µ) or RSL3 (4 µM) treatment for 24 h. H) Increased intracellular Fe^2^⁺ levels in PIR‐KO cells following erastin/RSL3 treatment. I) FerroOrange fluorescence imaging confirming Fe^2+^ accumulation in erastin‐treated PIR‐KO cells. Scale bar: 100 µm. J, K) Ectopic PIR overexpression in HCT15 cells validated via qPCR (J) and immunoblotting (K). L) Cellular viability in PIR‐overexpressing (OE) HCT15 cells following erastin (10 µm) or RSL3 (4 µm) treatment for 24 h. M) Attenuated MDA production in *PIR*‐OE versus WT cells under ferroptotic challenge. N) Flow cytometric assessment of lipid peroxidation using C11‐BODIPY staining in stably *PIR*‐OE HCT15 cells after erastin (10 µm) or RSL3 (4 µm) treatment for 24 h. O) Intracellular Fe^2+^ concentrations in WT and PIR‐OE HCT15 cells following erastin or RSL3 exposure. Data are presented as mean ± SD. ns, not significant, ^**^ *P*<0.01, ^***^ *P*<0.001, ^****^ *P*<0.0001, by 2‐tailed unpaired Student's *t*‐test (A and J), by 2‐way ANOVA with multiple comparisons (C, D, F–H, and L–O).

To validate the involvement of PIR in the modulation of ferroptosis, PIR expression was assessed in eight CRC cell lines using quantitative PCR (Figure S1A, Supporting Information). HCT15 and HCT116 cells, which showed elevated PIR expression, and HT1080 fibrosarcoma cells, a well‐established model for ferroptosis, were further investigated. CRISPR‐mediated ablation of PIR was confirmed by qPCR (**Figure** **2**A; Figure S1B, C, Supporting Information) and western blotting (Figure 2B; Figure S1D, Supporting Information). The cells were treated with erastin (a system x_c_^−^ inhibitor) or RSL3 (a GPX4 inhibitor) to induce ferroptosis.

Consistent with bioinformatic predictions (Figure 1), CCK‐8 assays demonstrated that PIR‐deficient HCT15 (Figure 2C–E), HCT116, and HT1080 cells (Figure S1E–I, Supporting Information) exhibited enhanced ferroptosis sensitivity to both inducers. Assessment using the BODIPY‐C11 probe revealed that PIR deficiency markedly increased erastin/RSL3‐induced lipid peroxidation (Figure 2F; Figure S1J, K, Supporting Information) and enhanced MDA accumulation, a key final product of lipid peroxidation, following treatment with erastin or RSL3 in HCT15 (Figure 2G), HCT116, and HT1080 cells (Figure S1L,M, Supporting Information). PIR‐deficient cells showed elevated intracellular Fe^2+^ levels compared with wild‐type cells after FINs treatments (Figure 2H,I; Figure S1N, O, Supporting Information). Conversely, PIR overexpression in HCT15 (Figure 2J,K) and HT1080 cells (Figure S1P, Q, Supporting Information) conferred robust FIN resistance (Figure 2L; Figure S1R, S, Supporting Information) and reduced ferroptosis indicators including MDA levels (Figure 2M; Figure S1T, Supporting Information), lipid peroxidation (Figure 2N; Figure S1U, Supporting Information), and Fe^2+^ accumulation (Figure 2O; Figure S1V, Supporting Information). These findings demonstrate PIR's functional role in mitigating ferroptotic vulnerability in CRC.

### 2.3 PIR is a Suppressor of Ferroptosis in CRC

To characterize the mechanism of PIR‐regulated cell death resistance, PIR‐deficient HCT15, HCT116, and HT1080 cells were treated with erastin or RSL3, and with various cell death inhibitors. PIR deletion exacerbated erastin‐ and RSL3‐mediated growth suppression in HCT15 (Figure S2A,B, Supporting Information), HCT116 (Figure S2C,D, Supporting Information), and HT1080 (Figure S2E,F, Supporting Information) cells. This phenotype was completely rescued by the ferroptosis inhibitor Fer‐1 and the iron chelator deferoxamine (DFO), but was unaffected by Nec‐1 (necroptosis inhibitor), 3‐MA (autophagy inhibitor), and Z‐VAD‐FMK (apoptosis inhibitor). This confirmed PIR's selective role in ferroptosis regulation. Pharmacological PIR inhibition using Triphenyl Compound A (TPhA) recapitulated the effects of genetic ablation: TPhA markedly enhanced erastin‐ and RSL3‐induced cytotoxicity (Figure S2G–I, Supporting Information), an effect that was completely reversed by Fer‐1. Genetic complementation via exogenous PIR expression in PIR‐KO HCT15, HCT116, and HT1080 cells (Figure S2J, Supporting Information) completely abrogated erastin‐ and RSL3‐driven increases in lipid peroxidation (Figure S2K‐M, Supporting Information) and cell death (Figure S2N–P, Supporting Information). These orthogonal genetic and pharmacological approaches have collectively established that PIR is a direct modulator of ferroptotic susceptibility in CRC models.

### 2.4 PIR Deficiency Sensitizes CRC to FINs In Vivo

**Figure 3:**
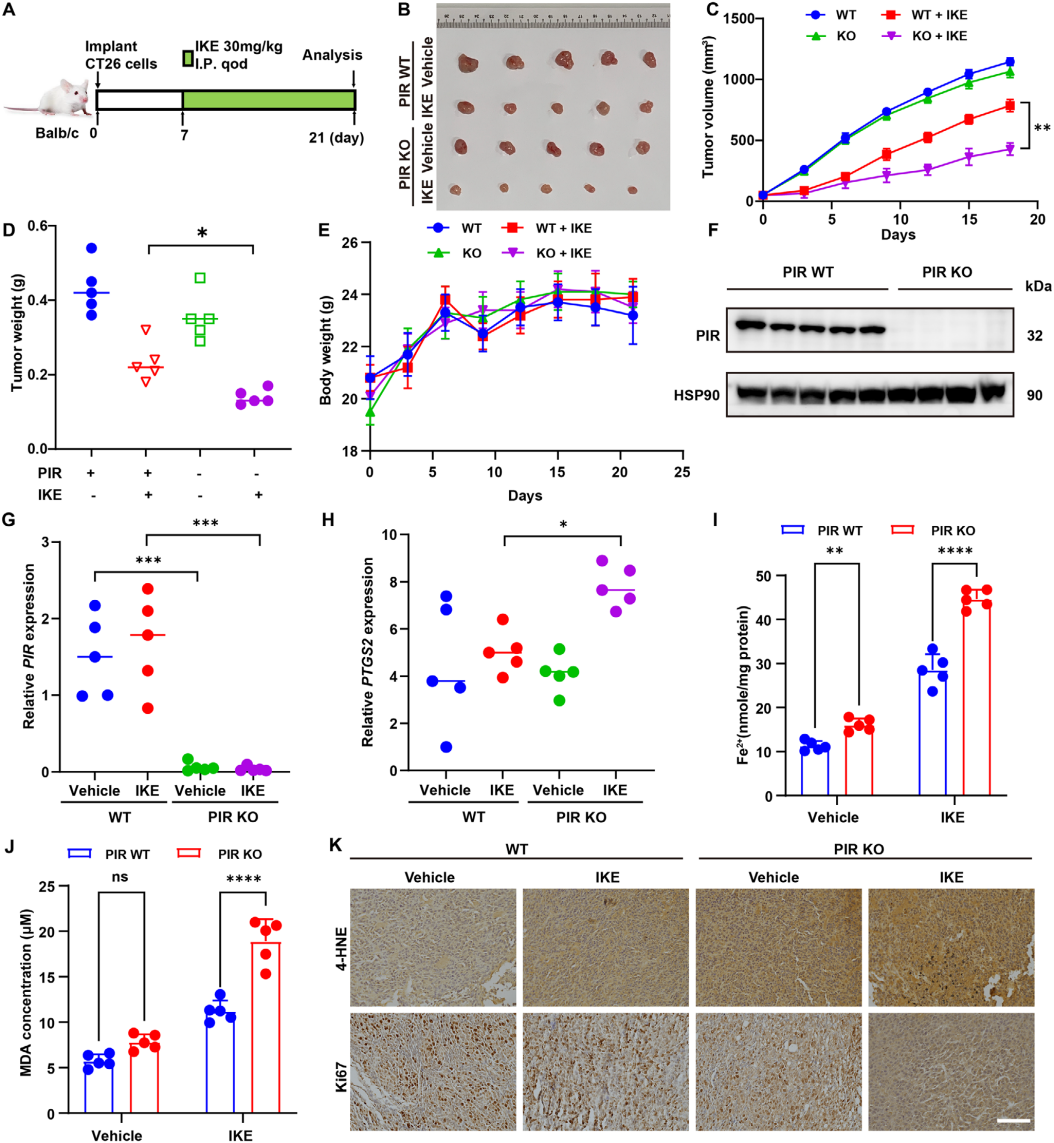
PIR attenuates ferroptotic cell death in CRC in vivo. A) Schematic of subcutaneous xenograft model and treatment protocol. BALB/c mice bearing CT26 WT and PIR‐ KO subcutaneous xenografts (n = 5 per group) received vehicle or imidazole ketone erastin (IKE; 30 mg kg^−1^, i.p.) on alternate days for 14 days. B) Macroscopic tumor specimens across experimental groups. C–E) Longitudinal monitoring of tumor volumes (C), terminal tumor masses (D), and body weights (E), across all groups (n = 5). F) Representative immunoblot of PIR protein expression in xenograft tumors. G, H) qRT‐PCR quantification of *PIR* (G) and *PTGS2* (H) transcripts in excised tumors. I, J) Intracellular Fe^2+^ (I) and MDA (J) concentrations in neoplastic tissues at experimental endpoint (day 14 post‐treatment) (n = 5 mice/group). K) IHC analysis of 4‐HNE and Ki‐67 in xenograft tissues from indicated mice treated with or without IKE. Scale bar: 100 µm. Data are presented as mean ± SD. ^*^ *P*<0.05, ^**^ *P*<0.01, ^***^ *P*<0.001, ^****^ *P*<0.0001, by 2‐way ANOVA with multiple comparisons (C, E, I and J), by 1‐way ANOVA with Dunnett's test (D, G and H).

The in vivo effects of PIR deficiency on ferroptosis susceptibility in CRC were investigated. Xenograft models were generated via the subcutaneous implantation of CRISPR/Cas9‐engineered PIR‐KO CT26 murine colon adenocarcinoma cells into BALB/c mice (n = 5 per group; **Figure** **3**A). On day 7 post‐implantation, and every other day until day 21, the mice received intraperitoneal injections of 30 mg kg^−1^ IKE, a metabolically stable ferroptosis inducer.^[^ ^33^ ^]^ Tumor growth kinetics demonstrated significantly attenuated tumor progression in the PIR‐KO cohorts compared with the controls following IKE treatment, as evidenced by the reduced tumor morphology (Figure 3B), volumes (Figure 3C), and weights (Figure 3D). No intergroup differences in body weight were observed, indicating minimal systemic toxicity (Figure 3E). Immunoblot validation and qRT‐PCR confirmed sustained PIR ablation in the excised tumors (Figure 3F,G). Elevated intratumoral *PTGS2* (cyclooxygenase‐2) mRNA, Fe^2+^, and MDA levels were detected in PIR‐KO tumors (Figure 3H–J), indicating enhanced ferroptotic activity. Immunostaining revealed diminished Ki‐67 proliferation indices and increased 4‐HNE staining in PIR‐deficient tumors, with the most pronounced effects observed in the IKE‐treated group (Figure 3K). These data establish PIR deficiency as a potent sensitizer of ferroptosis‐driven tumor suppression in vivo and are mechanistically linked to the amplification of iron‐dependent oxidative cascades.

### 2.5 Enhanced Ferroptosis in PIR‐Null Intestinal Epithelial Cells Attenuates CRC

**Figure 4:**
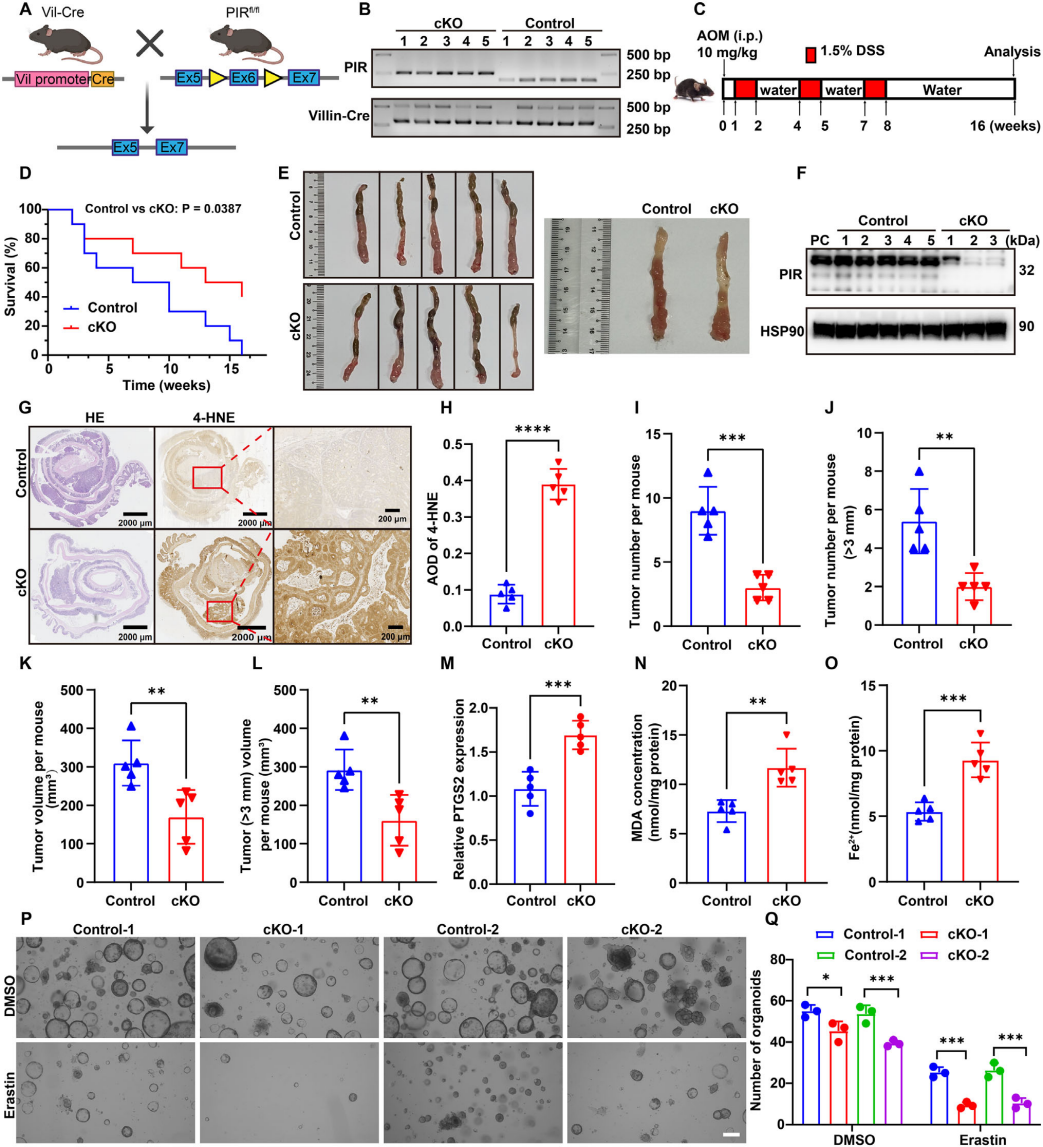
Epithelial PIR deletion enhances ferroptosis to suppress AOM/DSS‐induced tumorigenesis. A) Genetic strategy for *PIR*‐cKO murine model. B) Genotyping of *PIR^+/+^; Vil‐Cre* (control) and *PIR^fl/fl^; Vil‐Cre* (cKO) mice via PCR. C) AOM/DSS‐induced CRC timeline (i.p., intraperitoneal). D) Kaplan‐Meier survival analysis of control and cKO mice following AOM/DSS treatment (n = 10/group). E) Representative macroscopic colon specimens from AOM/DSS‐treated cohorts. F) PIR protein expression in neoplastic tissues from control and cKO models. G) H&E and 4‐HNE immunohistochemistry of colonic neoplastic tissues. Scale bar: 2000 and 200 µm. H) 4‐HNE staining quantification based on average optical density (AOD). I, J) Quantification of total tumor burden (I) and tumors exceeding 3 mm in diameter (J) per animal (n = 5 per group). K, L) Volumetric assessment of total tumor burden (K) and tumors greater than 3 mm (L) per mouse (n = 5 per group). M) *PTGS2* transcript levels in intestinal epithelia post‐AOM/DSS treatment. N, O) MDA concentration (N) and Fe^2+^ (O) quantification in colonic tumors of control and cKO cohorts. P) Microscopic images of tumor‐derived organoids obtained from control and cKO mice treated with vehicle or erastin. Scale bar: 200 µm. Q) Quantitative analysis of organoids for each experimental group shown in (P). Data are presented as mean ± SD. ^*^ *P*<0.05, ^**^ *P*<0.01, ^***^ *P*<0.001, ^****^ *P*<0.0001, by two‐tailed unpaired Student's *t*‐test (H‐O), by 2‐way ANOVA with multiple comparisons (Q).

We investigated the effect of PIR deficiency on CRC using conditional knockout (cKO) models and organoid systems. Villin‐Cre^ERT2^‐mediated recombination was used to generate PIR cKO mice in intestinal epithelial cells (IECs) (**Figure** **4**A). Genotypes were confirmed by PCR (Figure 4B). Oxidative stress resistance is a hallmark of tumorigenesis. Upregulated oxidative stress response pathways in human and murine tumors are associated with cancer stem cell proliferation and protect cells from reactive oxygen species (ROS)‐induced damage or cell death.^[^ ^34^, ^35^ ^]^ Despite these protective mechanisms, CRC epithelial cells exhibit a strong affinity for iron, which is required for tumor metabolism and growth, making them susceptible to ferroptosis under oxidative stress.^[^ ^36^, ^37^ ^]^

Previous AOM/DSS‐induced colitis‐associated cancer studies have shown that the inhibition of ferroptosis aggravates tumorigenesis,^[^ ^38^ ^]^ indicating that this model could be used to study ferroptotic regulation. To explore the role of PIR in mediating ferroptosis during CRC, we used an established AOM/DSS‐induced CRC mouse model. Control (*PIR^+/+^*; *Vil‐Cre*) and cKO (*PIR^fl/fl^*; *Vil‐Cre*) mice were treated with AOM/DSS following tamoxifen treatment (Figure 4C). The survival of PIR cKO mice was favorable (Figure 4D), and there was a clear reduction in tumor multiplicity (Figure 4E,I,J) and volume compared with the controls (Figure 4E,K,L). This indicated that PIR deficiency in IECs inhibited tumorigenesis. PIR protein levels were substantially reduced in cKO tumors (Figure 4F). H&E histopathological analysis revealed an attenuated tumor burden in cKO mice. Immunohistochemical profiling showed increased 4‐HNE, a marker of lipid peroxidation, in cKO tumors (Figure 4G,H), and tumor burden estimation verified the suppression in PIR‐deficient mice (Figure 4K,L). *PTGS2* expression, MDA levels, and ferrous iron content dramatically increased in PIR‐cKO tumors (Figure 4M–O), indicating the significance of PIR deficiency in fostering ferroptosis during the progression of CRC.

Tumor‐derived organoids from WT and PIR‐cKO mice were isolated to determine the regulatory roles of PIR in modulating ferroptosis. In comparison to the WT control group, the volume and viability of PIR cKO organoids subjected to erastin treatment were diminished (Figure 4P,Q), suggesting heightened sensitivity to ferroptosis. These findings indicate that PIR facilitates CRC growth through inhibiting ferroptosis. PIR deficiency enhances lipid peroxidation and ferroptosis, thereby impairing tumorigenesis in AOM/DSS‐driven models and ex vivo organoid systems.

### 2.6 Ferroptosis Inhibitors Reverse the Tumor‐Suppressive Effect of PIR Deficiency in CRC

To investigate the impact of PIR depletion‐induced lipid peroxidation in CRC, PIR cKO mice were treated with AOM/DSS followed by the administration of Lipro‐1, a lipophilic radical‐trapping ferroptosis inhibitor (Figure S3A, Supporting Information). Lipro‐1 treatment markedly restored the tumorigenic potential, as evidenced by decreased survival (Figure S3B, Supporting Information) and increased tumor multiplicity and volume compared with vehicle‐treated cKO mice (Figure S3C–G, Supporting Information). qPCR confirmed sustained *PIR* loss across cohorts (Figure S3H, Supporting Information). While PIR deficiency diminished the AOM/DSS‐induced tumor burden, including reduced incidence and fewer lesions >3 mm, Lipro‐1 restored tumor numbers to control levels (Figure S3D, E, Supporting Information). Biochemical assays showed that Lipro‐1 reversed ferroptosis‐associated perturbations from PIR loss and significantly reduced elevated 4‐HNE, *PTGS2*, MDA, and Fe^2^⁺ levels (Figure S3I–M, Supporting Information). Lipro‐1 co‐treatment completely blocked erastin‐induced growth suppression in PIR‐deficient organoids (Figure S3N, O, Supporting Information). These data indicate that PIR deletion suppresses CRC progression by potentiating ferroptosis, a phenotype that can be reversed by ferroptosis inhibition.

### 2.7 PIR Inhibition Augments Ferroptotic Sensitivity in Patient‐Derived CRC Models

The functional relevance of PIR in ferroptosis regulation was further validated using CRC patient‐derived cellular models. A comprehensive preclinical evaluation was conducted using primary patient‐derived cells (PDCs) and organoids (PDOs) cultured from treatment‐naïve CRC specimens to circumvent cell line limitations. Two PDCs (CRC1414 and CRC1850) and two PDOs (PDO1 and PDO2) were used in the experiments. In the PDC models, short hairpin RNAs (shRNAs)‐mediated PIR knockdown via two independent constructs (qPCR confirmation: Figure S4A,F, Supporting Information), potentiated erastin‐ and RSL3‐mediated cytotoxicity in CRC1414 and CRC1850 PIR‐knockdown cells compared with the controls (Figure S4B,C,G,H, Supporting Information). PIR ablation increased ferroptotic lipid peroxidation following erastin/RSL3 exposure (Figure S4D,I, Supporting Information) and elevated MDA levels (Figure S4E,J, Supporting Information). PDOs established from CRC patients were allocated to four groups: control, erastin, TPhA, and TPhA + erastin co‐culture. After 7 days of incubation, pharmacological PIR inhibition with TPhA synergistically enhanced erastin‐induced cytotoxicity, as evidenced by reduced organoid number and morphological integrity compared with the monotherapy groups (Figure S4K,L, Supporting Information). These results established that PIR suppression sensitized human CRC cells to ferroptosis induction across multiple experimental platforms, demonstrating the translational potential of combinatorial therapeutic strategies targeting ferroptotic vulnerability in CRC.

### 2.8 PIR Upregulation is Mediated by the Transcriptional Regulation of NRF2 During Ferroptosis

Ferroptosis inducers such as erastin upregulated PIR expression at both the transcriptional and translational levels in a time‐dependent manner (Figure S5A–D, Supporting Information). In HCT15 and HCT116 cells, PIR expression peaked 24 h after erastin exposure, coinciding with oxidative stress activation, a key feature of CRC pathogenesis. Given the established role of NRF2 in antioxidant response element (ARE)‐driven gene networks linked to iron and lipid metabolism, we investigated its effect on PIR. Treatment of CRC cells with tert‐butylhydroquinone (TBHQ) resulted in significant upregulation of the glutamate‐cysteine ligase modifier subunit (GCLM) and the glutamate‐cysteine ligase catalytic subunit (GCLC) (Figure S5E, F, Supporting Information). These results confirm the successful activation of the NRF2 pathway. Notably, PIR was significantly upregulated at both the transcriptional (Figure S5G, H, Supporting Information) and translational levels (Figure S5I, J, Supporting Information) in TBHQ‐treated HCT15 and HCT116 cells.

ChIP‐qPCR analysis directly confirmed that PIR was transcriptionally regulated by NRF2. Endogenous NRF2 occupancy was detected under basal conditions using primers targeting ARE in the PIR promoter. Erastin treatment significantly stimulated the binding of NRF2 to the PIR promoter, which is concurrent with the increased occupancy of established NRF2 targets, including heme oxygenase 1 (HMOX1) and NAD(P)H quinone dehydrogenase 1 (NQO1) (Figure S5K, L, Supporting Information). Collectively, these findings demonstrate that NRF2 is a key transcriptional regulator of PIR and establish a mechanistic link between oxidative stress adaptation and ferroptosis modulation in CRC.

### 2.9 PIR Inhibition Enhances Ferroptotic Susceptibility in CRC Cells via the Downregulation of PLA2G4A

**Figure 5:**
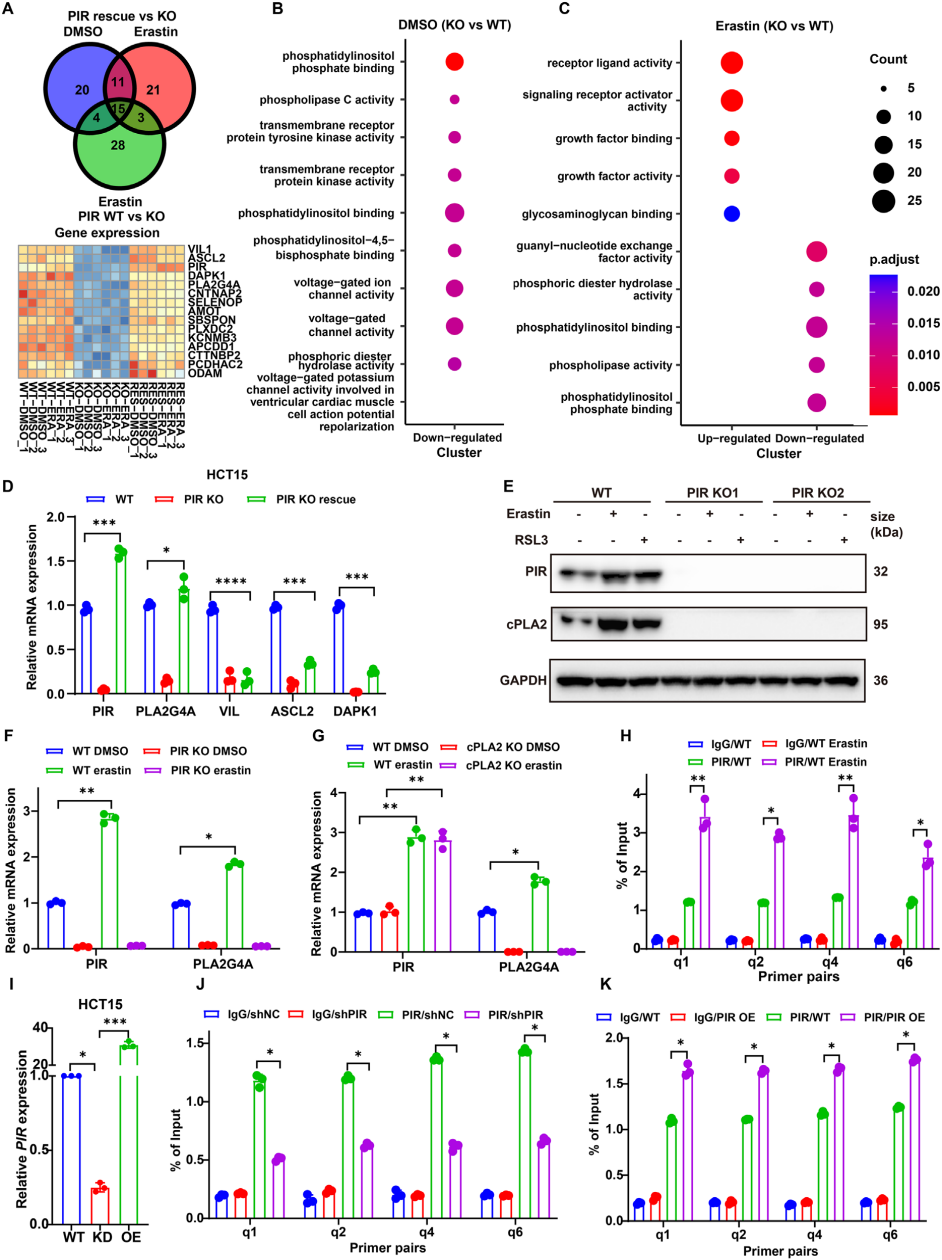
PLA2G4A mediates PIR‐dependent ferroptosis suppression. A) Venn diagram and heatmap of RNA‐seq data from PIR‐KO and PIR‐rescued HCT15 cells treated with erastin (10 µm, 24 h). B) GO analysis of molecular functions in PIR‐KO versus WT HCT15 cells. C) Bubble plot of molecular function alterations between HCT15 PIR‐KO and WT cells post‐erastin. D) qRT‐PCR of *PIR*, *PLA2G4A*, *VIL*, *ASCL2*, and *DAPK1* transcripts in PIR‐modulated HCT15 cells. E) Immunoblot of PIR and cPLA2 in erastin/RSL3‐treated PIR‐KO cells. F) *PIR* and *PLA2G4A* transcript expression in HCT15 cells after erastin (10 µm, 24 h). G) *PIR* and *PLA2G4A* expression in HCT15 *PLA2G4A*‐KO cells treated with erastin (10 µm, 24 h). H) ChIP‐qPCR of HCT15 cells post‐erastin (24 h). I) *PIR* knockdown/overexpression validation in HCT15 cells via qPCR. J) ChIP‐qPCR of HCT15 cells transfected with shPIR lentivirus. K) ChIP‐qPCR analysis demonstrated that *PIR* overexpression enhanced its enrichment at the PLA2G4A promoter region. Data are presented as mean ± SD. ^*^ *P*<0.05, ^**^ *P*<0.01, ^***^ *P*<0.001, ^****^ *P*<0.0001, by 2‐way ANOVA with multiple comparisons (D, F‐H and J‐K), by 1‐way ANOVA with Dunnett's test (I).

The mechanism underlying PIR‐mediated ferroptosis resistance was investigated through transcriptional profiling in CRC models. RNA‐seq of isogenic HCT15 cells (WT, PIR ‐ KO, and PIR‐rescued) exposed to erastin‐induced ferroptotic stress revealed several differentially expressed genes, the expression of which required PIR (**Figure** **5**A). PLA2G4A was the most promising candidate because of its ability to release AA. PLA2G4A encodes a critical enzyme in the AA metabolic pathway and is responsible for phospholipid remodeling. Gene Ontology (GO) enrichment analysis of genes significantly downregulated upon PIR ablation (log2fold change < −1, *P* < 0.05) revealed significant downregulation of the phosphatidylinositol phosphate binding pathway and phospholipase activity (Figure 5B), which was amplified by erastin treatment (Figure 5C). qPCR showed a potent reduction in PLA2G4A expression following PIR loss, whereas PIR restoration in HCT15 cells rescued PLA2G4A expression (Figure 5D). Immunoblotting also demonstrated a strong reduction in cPLA2 protein levels following PIR depletion under both basal and ferroptosis‐inducing conditions (Figure 5E).

PIR was used as a redox sensor to assess the effects of ferroptosis inducers on PLA2G4A expression. Erastin increased the synthesis of both PIR and PLA2G4A transcripts in wild‐type cells, and PIR silencing abrogated this induction (Figure 5F). In contrast, PLA2G4A deletion had no reciprocal effect on PIR expression, and erastin maintained PIR induction under PLA2G4A‐deficient conditions (Figure 5G), indicating that PLA2G4A is a downstream effector of PIR in ferroptosis regulation. PIR has previously been characterized as a transcriptional cofactor for NFκB1, enhancing its DNA‐binding affinity and transcriptional activity.^[^ ^39^ ^]^ To determine whether PIR regulates PLA2G4A transcription, ChIP‐qPCR was performed using six primer sets targeting the PLA2G4A promoter. The four primer sets demonstrated significant PIR enrichment, and erastin further increased PIR occupancy at these regulatory elements (Figure 5H). Stable PIR‐knockdown and PIR‐overexpressing HCT15 cell lines were generated and confirmed using qPCR to further validate the PIR‐mediated transcriptional regulation of PLA2G4A (Figure 5I). PIR silencing significantly reduced promoter enrichment (Figure 5J), whereas PIR overexpression markedly enhanced PIR occupancy at the PLA2G4A promoter (Figure 5K), confirming that PIR directly binds to PLA2G4A and regulates its transcription.

### 2.10 PLA2G4A Functions as the Critical Effector in PIR‐Mediated Ferroptosis Resistance

To delineate PLA2G4A's functional necessity for ferroptosis induction, PLA2G4A was genetically reconstituted in PIR‐knockout HCT15, HCT116, and HT1080 cells. Successful restoration was validated at the translational (Figure S6A, Supporting Information) and transcriptional (Figure S6B–D, Supporting Information) levels. Although the PIR knockout sensitized both cell lines to erastin‐ and RSL3‐induced cytotoxicity, PLA2G4A reconstitution in PIR‐deficient HCT15, HCT116 and HT1080 cells (Figure S6E‐G, Supporting Information) rescued cell viability and reduced lipid peroxidation, as evidenced by the diminished MDA levels (Figure S6H‐J, Supporting Information) and attenuated PIR knockout‐associated ROS accumulation (Figure S6K–M, Supporting Information). Patient‐derived CRC1414 cells demonstrated that restoration of PLA2G4A (Figure S6N, Supporting Information) rescued PIR knockdown‐induced ferroptotic susceptibility (Figure S6O,P, Supporting Information). These data establish that PLA2G4A is the principal effector of PIR‐mediated ferroptosis resistance.

Genetic ablation studies were conducted to validate PLA2G4A's direct role in ferroptosis regulation. The CRISPR/Cas9‐mediated PLA2G4A knockout in HCT15 cells (Figure S7A, B, Supporting Information) and shRNA knockdown in HT1080 cells (Figure S7C, D, Supporting Information) significantly potentiated erastin‐ and RSL3‐induced ferroptosis. Pharmacological inhibition studies using AACOCF3, a potent cPLA2 inhibitor that forms a covalent bond with serine 228 at the enzyme's active site, recapitulated these genetic findings.^[^ ^40^ ^]^ AACOCF3 significantly enhanced erastin‐induced cell death in both HCT15 and HT1080 cells (Figure S7E, F, Supporting Information) and induced a moderate increase in ROS, which was further exacerbated by ferroptosis inducers (Figure S7G, H, Supporting Information). These approaches establish PLA2G4A as a non‐redundant modulator of ferroptosis thresholds, operating downstream of PIR to mitigate oxidative cascades. The PIR‐PLA2G4A axis represents a therapeutically active pathway that augments ferroptosis sensitivity in colorectal malignancies.

### 2.11 PLA2G4A Inhibition Synergizes with Ferroptosis Inducers to Suppress Tumor Growth In Vivo

We evaluated the therapeutic potential of combining PLA2G4A inhibition with ferroptosis induction using AACOCF3 and IKE in CT26 xenograft‐bearing BALB/c mice, which were randomized into four cohorts: vehicle control, AACOCF3 monotherapy, AACOCF3/IKE combination, and AACOCF3/IKE plus the ferroptosis inhibitor Lipro‐1.

The AACOCF3/IKE combination significantly reduced the tumor mass and volume compared with the controls (Figure S8A–C, Supporting Information). The tumor specimens showed elevated levels of ferroptosis markers (*PTGS2*, MDA, ferrous iron, and 4‐HNE) following the combination treatment (Figure S8D–F, J, Supporting Information). The biochemical analysis confirmed the inhibition of PLA2G4A through decreased phospholipase A2 activity, increased AA levels, and reduced PGE2 concentrations (Figure S8G–I, Supporting Information).

Lipro‐1 co‐administration significantly attenuated the antitumor efficacy of AACOCF3/IKE therapy, confirming that enhanced ferroptotic cell death mediated therapeutic effects. These findings demonstrate that pharmacological inhibition of PLA2G4A effectively potentiates IKE‐induced ferroptosis and anticancer efficacy in vivo, thereby reinforcing the essential function of the PIR‐PLA2G4A axis in modulating ferroptotic susceptibility in CRC.

### 2.12 AACOCF3 Attenuates CRC by Enhancing Ferroptotic Cell Death

**Figure 6:**
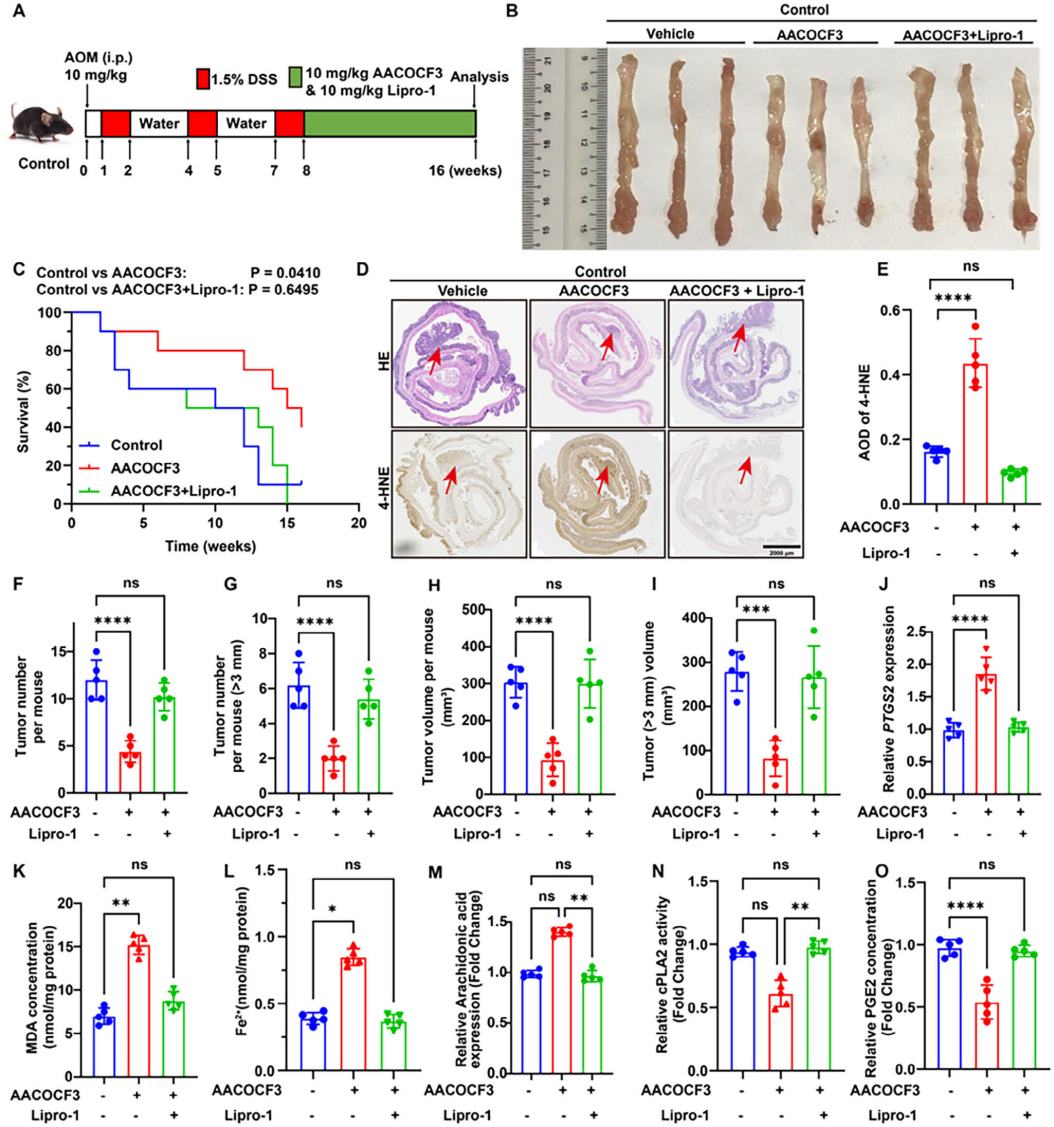
AACOCF3 attenuates AOM/DSS‐induced tumorigenesis. A) Experimental schema of AOM/DSS carcinogenesis in control mice treated with AACOCF3 (10 mg kg^−1^, i.p.) or Lipro‐1 (10 mg kg^−1^, i.p.). B) Macroscopic colorectal tissue from the indicated groups. C) Kaplan‐Meier analysis showing improved survival with AACOCF3 (*P* = 0.0410), abolished by Lipro‐1 (*P* = 0.6495). D) Histopathological analysis using H&E staining and immunohistochemical detection of 4‐HNE in neoplastic tissues. Scale bar: 2000 µm. E) Semi‐quantitative analysis of 4‐HNE expression determined by AOD measurements. F, G) Quantification of total tumor burden (F) and tumors exceeding 3 mm in diameter (G) per animal (n = 5 per group). H, I) Volumetric assessment of total tumor burden (H) and tumors greater than 3 mm (I) per mouse following various treatment regimens (n = 5 per group). J ‐ L) Biochemical analysis of colonic tumor tissue for *PTGS2* expression (J), MDA content (K), and iron concentrations (L) (n = 5 per group). M ‐ O) Quantitative determination of arachidonic acid levels (M), cPLA2 enzymatic activity (N), and PGE2 concentrations (O). Data are presented as mean ± SD. ^*^ *P*<0.05, ^**^ *P*<0.01, ^***^ *P*<0.001, ^****^ *P*<0.0001, by 1‐way ANOVA with Dunnett's test (panels E‐O).

Previous studies demonstrated that PIR deficiency impairs AOM/DSS‐induced CRC via lipid peroxidation‐dependent ferroptosis. Because PIR regulates ferroptotic susceptibility by modulating PLA2G4A. Pharmacological inhibition of cPLA2 represents a rational therapeutic approach. In this study, we evaluated the effectiveness of AACOCF3 in an autochthonous AOM/DSS CRC model. Colitis‐associated CRC was induced using the AOM/DSS protocol, followed by the administration of vehicle control or AACOCF3 (10 mg kg^−1^), with or without the ferroptosis inhibitor Lipro‐1 (10 mg kg^−1^; **Figure** **6**A). AACOCF3 monotherapy demonstrated significant anticancer efficacy, decreasing tumor quantity and size while extending overall survival (Figure 6B–G). Notably, this tumor‐suppressive effect was completely abolished by the co‐administration of Lipro‐1, as the survival and tumor burden levels were comparable to those of the controls.

Immunohistochemical analysis showed significantly elevated 4‐HNE levels in neoplastic tissues from AACOCF3‐treated subjects (Figure 6D,E). Concomitantly, additional ferroptosis markers, including *PTGS2* mRNA expression (Figure 6J), MDA content (Figure 6K), and ferrous iron accumulation (Figure 6L), were significantly increased following AACOCF3 treatment. These molecular alterations were substantially attenuated by co‐treatment with Lipro‐1 (Figure 6D–L).

Metabolomic analyses, measured by cPLA2α’s role in AA release and PGE2 biosynthesis, revealed that AACOCF3 administration significantly elevated free AA concentrations while suppressing cPLA2 enzymatic activity and reducing PGE2 levels. Lipro‐1 co‐administration effectively reversed these metabolic perturbations (Figure 6M–O). These data collectively established that AACOCF3 exerted its antitumor effects in the AOM/DSS model by enhancing lipid peroxidation‐dependent ferroptosis, highlighting its therapeutic potential for clinical translation in CRC.

### 2.13 PIR Drives Ferroptosis‐Modulating Lipidome Reprogramming in CRC

**Figure 7:**
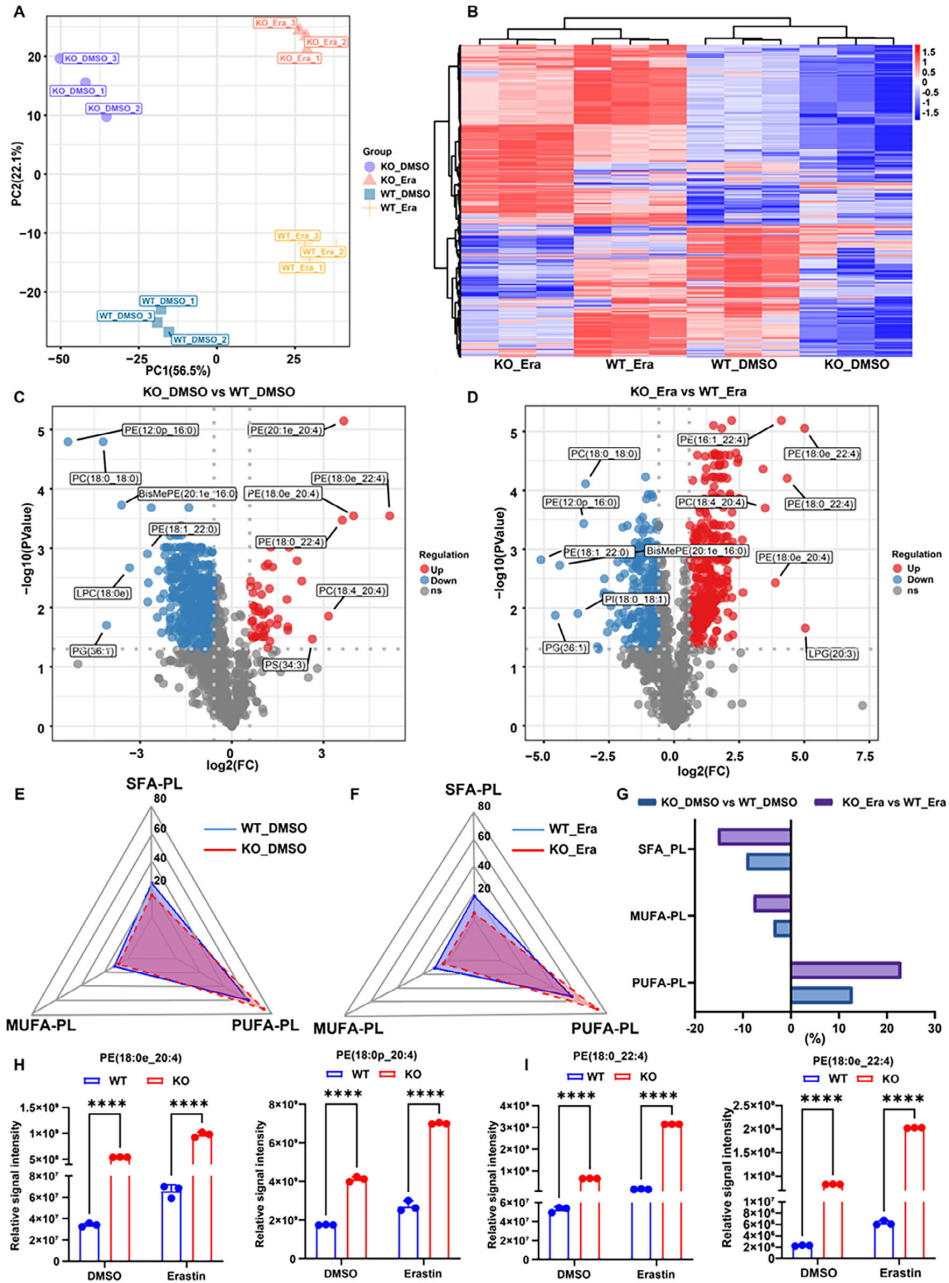
Untargeted lipidomics reveals PIR deletion‐mediated lipid metabolic remodeling. A) Principal component analysis (PCA) of global lipid profiles in HCT15 WT and *PIR*‐KO cells treated with or without erastin. B) Heatmap of differentially abundant lipid species in PIR‐KO versus WT cells with or without erastin treatment. C) Volcano plot of altered phospholipids (|log2 FC| >1, ^*^ *P* < 0.05) in PIR‐KO versus WT cells; upregulated (red), non‐significant (grey), downregulated (blue). D) Volcano plot of erastin‐induced phospholipid changes in PIR‐KO versus WT cells. E) Radar chart of SFA‐, MUFA‐, and PUFA‐containing phospholipids in WT and PIR‐KO cells. F) Radar chart of erastin‐modulated SFA‐, MUFA‐, and PUFA‐PLs distributions. G) Relative changes in phospholipid subclasses. H, I) Arachidonic acid (AA; H) and adrenic acid (AdA; I) intensities in WT and PIR‐KO cells treated with or without erastin. Data are presented as mean ± SD. ^****^ *P*<0.0001, by 2‐way ANOVA with multiple comparisons (H and I).

cPLA2, encoded by PLA2G4A, is a critical enzyme that hydrolyzes membrane phospholipids to release AA for eicosanoid biosynthesis. Given AA's role in ferroptosis, a global lipidomic analysis was conducted using PIR‐knockout and wild‐type HCT15 cells treated with erastin or vehicle for 24 h. Principal component analysis (PCA) revealed clear separation and distinct clustering between the experimental groups (**Figure** **7**A). Differential phospholipid (PL) analysis revealed significant alterations in phospholipid abundance in PIR‐deficient cells compared with wild‐type controls, with erastin treatment further amplifying these lipidomic shifts (Figure 7B–D). Ferroptotic execution is primarily mediated by oxidized phospholipids containing PUFAs, whereas MUFAs and SFAs protect against ferroptosis.^[^ ^41^, ^42^ ^]^

LC‐MS‐based lipidomic analysis demonstrated significant reductions in MUFA‐ and SFA‐containing phospholipids, particularly phosphatidylcholines (PCs) and phosphatidylethanolamines (PEs), accompanied by an increase in PUFA‐containing phospholipids in both wild‐type and PIR‐knockout cells (Figure 7E,F). Detailed phospholipid analysis revealed that PIR deficiency significantly increased the proportion of PUFA‐containing phospholipids while reducing MUFA and SFA levels (Figure 7E). Erastin treatment further potentiated these compositional alterations (Figure 7F,G). Further examination of ferroptosis‐related lipid species demonstrated that PIR knockout elevated the levels of AA‐containing phospholipids, including PE (18:0e_20:4) and PE (18:0p_20:4), as well as adrenic acid (AdA)‐containing species PE (18:0_22:4) and PE (18:0e_22:4), which were further enhanced upon erastin exposure (Figure 7H,I).

These lipidomic alterations underscore the critical balance between MUFA‐ and PUFA‐containing phospholipids in determining susceptibility to ferroptosis.^[^ ^43^ ^]^ Remodeling suggested that PIR‐deficient cells possessed a heightened vulnerability to ferroptotic lipid peroxidation. Interestingly, despite the increased sensitivity of PIR‐knockout cells, not all PUFA‐containing phospholipids actively contributed to ferroptosis.

### 2.14 Clinical Relevance of the PIR‐PLA2G4A Pathway in Human CRC

**Figure 8:**
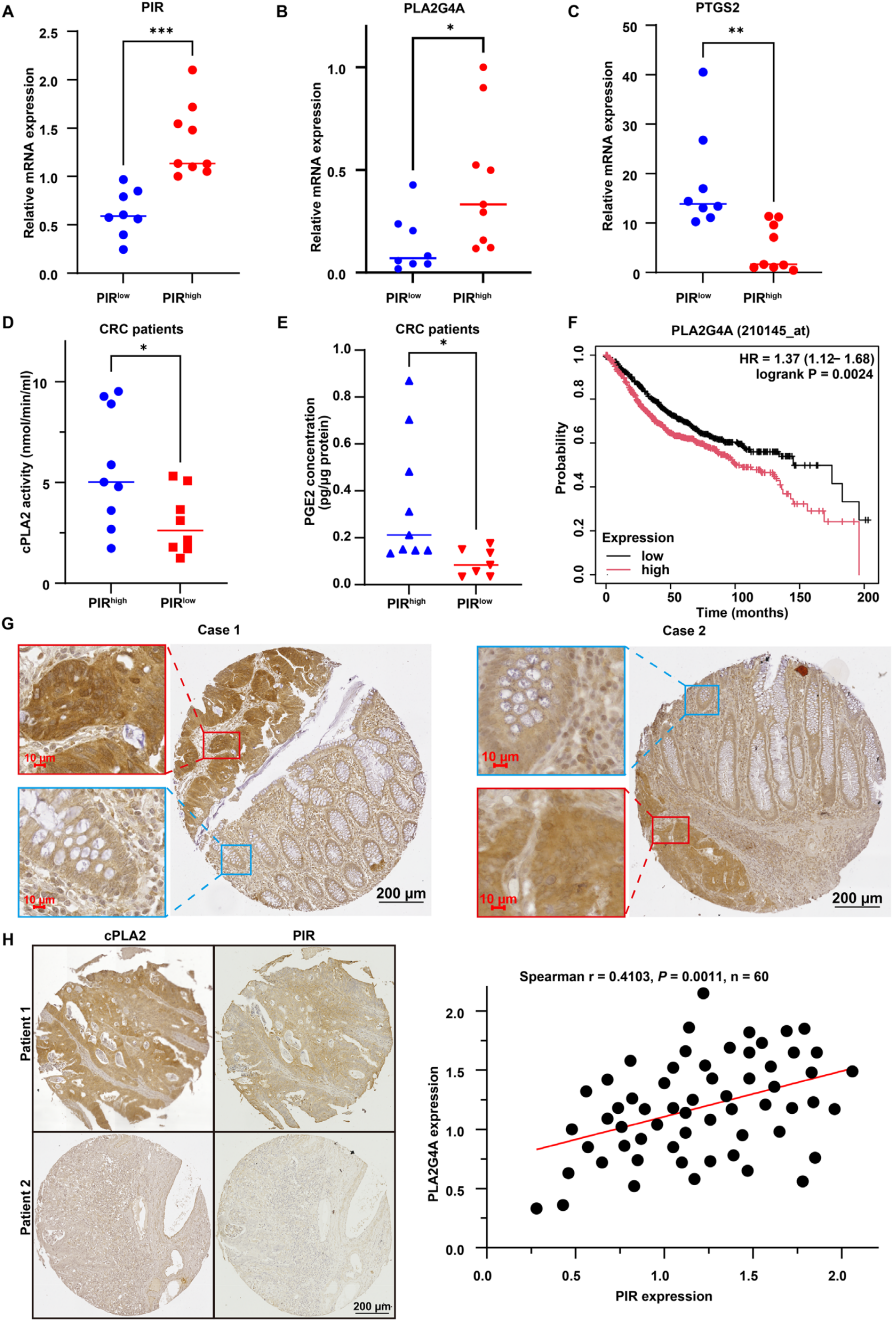
PLA2G4A is upregulated in CRC tissues and correlates with PIR expression. A) qRT‐PCR analysis of *PIR* transcripts in CRC clinical specimens. B, C) PLA2G4A (B) and PTGS2 (C) in PIR^low^ versus PIR^high^ CRC patient cohorts. D, E) cPLA2 enzymatic activity (D) and PGE2 concentrations (E) in PIR^low^ and PIR^high^ tumor tissues. F) Kaplan Meier survival analysis of PLA2G4A high expression in CRC (log‐rank test). G) Representative immunohistochemistry of PLA2G4A expression in CRC TMA specimens. H) Co‐staining analysis of PIR and PLA2G4A in CRC patient‐derived TMA sections showing coordinate expression patterns. Data are presented as mean ± SD. *P<0.05, **P<0.01, ***P<0.001, by 2‐tailed unpaired Student's *t*‐test (A‐E).

To investigate the clinical relevance of PIR‐mediated PLA2G4A regulation in CRC, we analyzed PIR and PLA2G4A expression in paired neoplastic and adjacent non‐neoplastic tissues from 17 patients with CRC. The specimens were stratified into PIR^high^ and PIR^low^ cohorts based on quantitative PIR mRNA expression analysis (**Figure** **8**A). The PIR^high^ cohort showed elevated PLA2G4A mRNA expression compared with the PIR^low^ specimens (Figure 8B). Conversely, PTGS2 expression was significantly upregulated in PIR^low^ specimens, indicating increased ferroptotic activity (Figure 8C). Furthermore, PIR^high^ specimens exhibited markedly increased cytosolic phospholipase A2 activity and PGE2 concentration (Figure 8D, E). Kaplan‐Meier analysis revealed that diminished PLA2G4A expression was correlated with improved clinical outcomes in patients with CRC (Figure 8F). Immunohistochemical analysis further confirmed significantly elevated PLA2G4A expression in neoplastic tissues compared with adjacent non‐neoplastic tissues (Figure 8G). A positive correlation between PLA2G4A and PIR expression was observed in the CRC specimens (Figure 8H). Collectively, these clinical data align with the preclinical evidence demonstrating that PIR‐driven PLA2G4A activation suppresses ferroptosis, thereby promoting CRC development. The translational relevance of this axis makes it a promising therapeutic target for ferroptosis‐based CRC treatments.

## 3 Discussion

Ferroptosis is a pivotal tumor suppressive mechanism in gastrointestinal malignancies, including CRC.^[^ ^44^ ^]^ Since CRC cells frequently exhibit resistance to apoptosis, alternative regulated cell death pathways, such as ferroptosis, represent attractive therapeutic targets. However, molecular regulators of ferroptotic susceptibility in CRC pathogenesis have not yet been reported. This study identifies PIR as a critical ferroptosis suppressor that functions through lipid membrane remodeling, revealing a targetable NRF2‐PIR‐PLA2G4A regulatory axis that governs ferroptosis susceptibility in CRC. Elevated PIR expression correlates with reduced ferroptosis sensitivity and aggressive tumor behavior, whereas PIR depletion consistently inhibits CRC progression across multiple experimental systems. The robust anti‐tumor effects observed in intestinal epithelial‐specific PIR‐knockout mice subjected to AOM/DSS‐induced carcinogenesis, characterized by increased lipid peroxidation and enhanced ferroptosis, demonstrate that PIR functions as a critical resistance factor in vivo. These findings suggest that PIR is a potential prognostic biomarker and therapeutic target in CRC.

PIR is a highly conserved member of the cupin superfamily, with orthologs in mammals, plants, fungi, and prokaryotes.^[^ ^45^ ^]^ As a transcriptional co‐factor, PIR interacts with nuclear I/CCAAT box transcription factor NFI/CTF1 and BCL‐3, potentiating NFκB signaling^[^ ^39^ ^]^ and promoting malignancy.^[^ ^46^ ^]^ While PIR's role in multiple tumors has been documented, its regulatory function in ferroptosis in CRC remains unexplored. This work has identified a compensatory NRF2‐PIR feedback circuit that confers adaptive resistance to ferroptosis.

We identified NRF2 as an upstream regulator of PIR expression during ferroptotic stress, which is consistent with its established role in redox homeostasis.^[^ ^47^ ^]^ Under ferroptotic stress, NRF2 activation drives PIR transcription via direct promoter binding, thereby establishing a cytoprotective response that attenuates ferroptotic cell death. NRF2 is a master regulator of cellular antioxidant response. Under basal conditions, NRF2 protein levels remain low owing to KEAP1‐mediated ubiquitin‐proteasome degradation. Upon oxidative or electrophilic stress, NRF2 stabilization enables nuclear accumulation, where it heterodimerizes with small musculoaponeurotic fibrosarcoma (sMAF) proteins and binds to AREs in target gene promoters, thereby inducing the expression of diverse detoxification and antioxidant enzymes.^[^ ^48^ ^]^ While NRF2 activation protects normal cells from oxidative damage, its constitutive overexpression in malignant cells confers drug resistance by augmenting antioxidant defense and suppressing ferroptosis.^[^ ^49^ ^]^ Although NRF2‐mediated ferroptosis resistance through classical targets, including GPX4,^[^ ^50^ ^]^ FSP1,^[^ ^51^ ^]^ and SLC7A11,^[^ ^52^ ^]^ is well established, the requirement for individual target genes in NRF2's anti‐ferroptotic function remains unclear. Our identification of PIR as an NRF2 effector reveals an additional mechanism of adaptive ferroptosis resistance. Erastin treatment enhances NRF2 binding to the PIR promoter, increasing PIR expression and establishing a compensatory circuit that limits the therapeutic efficacy of ferroptosis‐inducing agents. This adaptive mechanism likely explains the modest benefit of NRF2 inhibition monotherapy, suggesting that combinatorial targeting of multiple NRF2‐regulated pathways, including PIR, may be necessary to effectively overcome ferroptosis resistance. While ferroptosis inducers initially trigger oxidative stress and lipid peroxidation, concurrent activation of the NRF2‐PIR axis promotes resistance through PLA2G4A‐mediated lipid remodeling.

The anti‐ferroptotic activity of PIR occurs through the transcriptional upregulation of PLA2G4A, which encodes cPLA2, a critical lipid‐remodeling enzyme. PIR deficiency transcriptionally suppresses PLA2G4A, impairing arachidonic acid metabolism and altering the cellular lipidome. The Lands cycle constitutes an essential phospholipid remodeling pathway, in which PLA2 hydrolyzes the sn‐2 ester bond in membrane phospholipids, generating lysophospholipids, which are subsequently acylated by lysophospholipid acyltransferase, thereby incorporating new fatty acids.^[^ ^53^ ^]^ By removing oxidized PUFA‐containing phospholipids, PLA2 provides a critical defense against ferroptosis,^[^ ^25^, ^54^ ^]^ although its activity under oxidative stress exhibits context‐dependent complexity. cPLA2 mediates calcium‐dependent phospholipase and lysophospholipase activities, governing membrane remodeling and the biosynthesis of bioactive lipid mediators. While cPLA2 undergoes complex transcriptional regulation^[^ ^55^ ^]^ by cytokines and growth factors,^[^ ^56^ ^]^ our findings identified PIR as a previously unrecognized regulator of PLA2G4A through direct promoter binding.

The regulatory role of cPLA2 in ferroptosis is mechanistically complex and context‐dependent, and emerging evidence has revealed paradoxical, tissue‐specific effects. cPLA2‐driven ferroptosis has been implicated in heatstroke‐induced liver injury^[^ ^57^ ^]^ and thrombin‐activated cPLA2α‐mediated AA release triggering ferroptosis in triple‐negative breast cancer.^[^ ^58^ ^]^ Conversely, in prostate cancer, ATF6α‐driven PLA2G4A activation promotes PGE2 synthesis to suppress ferroptosis.^[^ ^59^ ^]^ This study demonstrates that the PIR knockout reduced PLA2G4A expression, attenuated cPLA2 enzymatic activity, decreased PGE2 levels, and elevated AA concentrations. PLA2G4A activation initiates phospholipid hydrolysis, liberates AA, and generates PGE2, a critical mediator of inflammatory cascades and oncogenic progression.^[^ ^60^ ^]^ PGE2 synergizes with N‐acetylcysteine to inhibit hemin‐induced ferroptosis.^[^ ^61^ ^]^ PGE2 also protects against cerebral ischemia/reperfusion‐induced ferroptosis, potentially via PGE2 receptor subtypes 3 and 4.^[^ ^62^ ^]^ Consequently, PIR transcriptionally regulates PLA2G4A, modulating downstream metabolite production and thereby influencing ferroptotic susceptibility.

The functional importance of the PIR‐PLA2G4A axis is supported by therapeutic intervention studies. These studies demonstrate that pharmacological inhibition of PLA2G4A with AACOCF3 or genetic disruption of PIR‐PA2G4A signaling synergizes with ferroptosis inducers to suppress CRC growth in preclinical models. AACOCF3, a cell‐permeable arachidonic acid analog containing a trifluoromethyl ketone moiety, functions as a potent, selective, slow‐binding cPLA2 inhibitor.^[^ ^63^, ^64^ ^]^ Pharmacological validation using AACOCF3 provides translational evidence supporting the idea that targeting the PIR‐PLA2G4A axis in CRC therapy demonstrates pathway druggability and establishes proof‐of‐concept for PLA2G4A inhibition as a strategy to overcome ferroptosis resistance. This combinatorial approach effectively dismantles adaptive resistance mechanisms and sensitizes tumors to ferroptosis induction. The clinical availability of PLA2G4A inhibitors with established safety profiles has enhanced their translational potential. Future investigations should optimize dosing regimens, identify patient populations likely to benefit from PIR/PLA2G4A expression profiles, and evaluate whether this combination strategy overcomes resistance to conventional chemotherapy or targeted therapies in CRC.

Ferroptosis is mechanistically linked to membrane phospholipid synthesis and lipid peroxidation, and cellular susceptibility is determined by lipid remodeling kinetics. The equilibrium between MUFA‐ and PUFA‐containing phospholipids critically governs ferroptotic sensitivity^[^ ^43^ ^]^ by generating peroxidation‐prone substrates^[^ ^65^ ^]^ with PE and PC serving as the primary ferroptosis executors.^[^ ^66^, ^67^ ^]^ Given cPLA2's role in lipid homeostasis, we hypothesized that PIR regulates this pathway. Untargeted lipidomics revealed that the PIR knockout increased PUFA incorporation while reducing SFA and MUFA content in phospholipids. In particular, the PE effects were amplified under erastin‐induced ferroptotic stress. Ferroptotic cascades predominantly involve the peroxidation of AA‐enriched PE (e.g., PE 18:0_20:4), AdA‐containing PE (e.g., PE 18:0_22:4), and docosahexaenoic acid (DHA)‐incorporated PC species (e.g., PC 18:0_22:6).^[^ ^68^, ^69^ ^]^ Our analyses revealed extensive PC and PE subspecies remodeling, with prominent alterations in AA‐ (PE 18:0_20:4) and AdA‐containing species (PE 18:0_22:4). These findings indicate that the deletion of PIR substantially alters phospholipid composition and facilitates the progression of ferroptosis. Notably, our results contrast with previous reports on PLA2G4A. Although reduced cPLA2 activity predicts AA depletion, paradoxical AA accumulation occurs along with global lipidome reorganization. Considering the dual function of PLA2—cytoprotection against oxidative stress versus activation by oxidative stress, culminating in cellular damage—we propose that PIR deletion attenuates cPLA2 expression and activity, impairs cellular antioxidant defenses, and increases ferroptotic susceptibility. Despite reduced cPLA2 activity following PIR ablation, the PLA2 superfamily comprises several members that catalyze sn‐2 position hydrolysis with a potential functional interplay. Consequently, diminished cPLA2 activity may modulate other family members, causing lipid remodeling and elevated AA concentrations. However, the molecular mechanisms governing cPLA2 interactions with other PLA2 family members have not yet been fully characterized. This investigation demonstrates that PIR transcriptionally regulates PLA2G4A to modulate lipid metabolism, a finding derived primarily from cell line experiments. Future studies incorporating comprehensive patient‐derived lipidomics will be essential to elucidate the clinical implications and establish the potential of therapeutic biomarkers.

In conclusion, this study has defined a previously unrecognized NRF2‐PIR‐PLA2G4A regulatory circuit that governs ferroptotic susceptibility through coordinated lipidome remodeling in CRC. PIR functions as a critical node linking transcriptional stress responses to membrane lipid composition, establishing adaptive ferroptosis resistance. Disruption of this axis, either pharmacologically or genetically, sensitizes CRC cells to ferroptosis and synergizes with ferroptosis‐inducing agents, providing a mechanistic foundation for combination therapeutic strategies to overcome resistance and enhance the efficacy of ferroptosis‐based cancer therapy.

## 4 Experimental Section

Additional materials and methods are available in the Supporting Information.

### Patient Cohorts and Biospecimens

A retrospective cohort of 159 archival formalin‐fixed, paraffin‐embedded (FFPE) primary CRC specimens was acquired from patients receiving curative resection at Singapore General Hospital (SGH). Tissue microarrays (TMAs) were constructed for uniform IHC staining. Whole‐slide imaging of immunostained sections was performed using a Zeiss Axio Scan Z1 high‐throughput slide scanning platform (Carl Zeiss AG, Germany). Histopathological quantification via H‐scoring was conducted independently by two board‐certified pathologists under blinded conditions.

A prospective subset of 10 matched fresh tumor‐normal tissue pairs was procured from the National University Hospital (NUH) for the establishment of patient‐derived organoid (PDO) cultures and subsequent functional validation. All human biospecimens were obtained with written informed consent in accordance with protocols approved by the Institutional Review Boards of Singhealth, NUH, and the Institute of Molecular and Cell Biology (IMCB), A*STAR, in compliance with the Declaration of Helsinki guidelines.

### Animals

This investigation adhered to all applicable ethical guidelines for animal research. All murine experimental procedures and protocols were executed with the approval and supervision of the Institutional Animal Care and Use Committee at A*STAR (Singapore). BALB/c mice, aged 6 to 8 weeks, were used for xenograft tumor growth experiments. PIR‐floxed mice were generated at Cyagen Biosciences through targeted insertion of LoxP sites flanking exon 6 (85 bp) of the *Pir* gene, creating conditional knockout alleles. Villin‐Cre^ERT2 ]^transgenic mice were produced from Jackson Laboratories. Cre‐mediated recombination was induced by administering tamoxifen (2 mg per mouse, i.p., days 1–3). The maximum permissible tumor burden was limited to 2000 mm^3^ in accordance with institutional guidelines. Mice were housed under specific pathogen‐free (SPF) conditions with a 12‐h light‐dark cycle, regulated temperature (20–25 °C), humidity (40–60%), and a normal diet. Veterinary staff routinely monitored health status. Euthanasia was performed via CO_2_ asphyxia at experimental endpoints, followed by tissue collection.

### ROS Detection

Cells were plated at a density of 1 × 10^5^ cells per well in 12‐well plates in technical triplicate. After being exposed to DMSO or specific drugs, cells were treated with 2 µm BODIPY 581/591 C11 lipid peroxidation sensor (Invitrogen, D3861) at 37 °C for 30 min. Subsequently, the labelled cells were rinsed twice with phosphate‐buffered saline (PBS), detached by trypsinization, and then resuspended in cold PBS to make a single‐cell suspension. Before analysis, the suspensions were filtered through a 40‐µm cell strainer. Lipid peroxidation was evaluated by measuring the fluorescence emission of oxidized C11 in the FITC channel with an LSRII flow cytometer (Becton Dickinson). A minimum of 5000 cellular events were acquired for each experimental condition across triplicate samples. Data analyses were conducted using FlowJo software (BD Biosciences).

### Iron Assay

The Intracellular iron concentration was detected by a commercial Iron Assay Kit (ab83366, Abcam) in accordance with the protocol of the manufacturer. Cells cultured in six‐well plates were homogenized in five volumes (v/v) of assay buffer. The resultant lysis buffer was centrifuged at 13000 × g for 15 min at 4 °C. The supernatant was obtained following the removal of insoluble debris. For iron quantification, 50 µL aliquots of each sample were treated with 5 µL of iron reducer to facilitate the conversion of ferric iron (Fe^3+^) to ferrous iron (Fe^2+^), followed by incubation at 25 °C for 30 min. Subsequently, 100 µL of iron probe was added to each sample and incubated at room temperature in the dark for 1 h. Absorbance was measured at 593 nm with a microplate spectrophotometer, and the iron concentration was determined using interpolation from a standard curve.

Intracellular Fe^2+^ was visualized by incubating cells with 5 µm FerroOrange (F374, Dojindo Laboratories) in complete medium for 30 min at 37 °C under dark conditions. The nuclei were subsequently counterstained with DAPI. Following PBS washing, fluorescence images were obtained using a confocal laser scanning microscope (LSM800, Zeiss).

### Chromatin Immunoprecipitation (ChIP)

Cells were cross‐linked on growth plates by gently agitating with 1.5 mm disuccinimidyl glutarate (DSG, Thermo Fisher Scientific) for 45 min at room temperature. Following DSG removal, cells were fixed with 1% formaldehyde for 10 min, after which glycine was added to achieve a final concentration of 0.125 m to terminate the reaction. Cells were lysed and then sonicated to produce chromatin fragments averaging 150–600 bp in size. The lysate was pre‐cleared with Protein A/G Sepharose beads for 1 h. Following centrifugation, 10% of the supernatant was reserved as the input sample. ≈4 million cells’ chromatin was treated overnight at 4 °C with 4 µg of NRF2 antibody (Abcam, ab137550) or PIR antibody (Abcam, ab227280). Immunocomplexes were precipitated with ChIP‐grade protein A/G magnetic beads (26162, Life Technology), followed by overnight reverse cross‐linking, and the sample was eluted to a final volume of 60 µL. ChIP‐qPCR utilizing primers specific to the regions of PIR or PLA2G4A. Enrichment was assessed using the 10% input method.

### MDA Measurement

Intracellular malondialdehyde (MDA) concentrations were quantitatively assessed using a Lipid Peroxidation (MDA) Assay Kit (MAK085, Sigma) in accordance with the manufacturer's guidelines. Cells were inoculated in 6‐well plates at a density of 5 × 10^4^ cells per well and permitted to adhere overnight under standard culture conditions. After treating cells with the specific compound at specific time intervals, cells were lysed using the MDA lysis solution included in the kit. The cell lysates were subsequently incubated with thiobarbituric acid (TBA) reagent to facilitate the formation of MDA‐TBA adducts through a condensation reaction. The MDA‐TBA chromogenic complex was quantified spectrophotometrically using a TECAN Spark 10 m microplate reader (TECAN Group Ltd., Switzerland) at 532 nm. The MDA concentration was determined by comparing it with a standard curve generated using MDA standards with known concentrations.

### RNA Sequencing

RNA sequencing (RNA‐seq) was performed by Novogene. Total RNA was extracted from three replicate samples of HCT15 cells under different treatment conditions, as well as from HCT15 PIR KO cells and HCT15 PIR rescue cells, using the TRIzol reagent (No. 15 596 018, Thermo Fisher Scientific). The quality of RNA was evaluated via 1% agarose gel electrophoresis, and its integrity was analyzed using the RNA Nano 6000 Assay Kit on a Bioanalyzer 2100 machine (Agilent Technologies). RNA‐seq libraries were constructed utilizing the NEBNext Ultra II directional RNA library preparation kit (NEB) in accordance with the manufacturer's protocol. FastQC v0.11.8 and Trimmomatic v0.321 were employed to filter raw reads. The processed reads were aligned to the human genome (hg38) with STAR v2.7.10b. The DESeq2 R package was used for differential expression analysis. Significantly differentially expressed genes (DEGs) were defined as genes with an absolute fold change ≥ 2 and a false discovery rate (FDR) < 0.05. Further analyses, including functional grouping and pathway enrichment analysis, were performed in R using the fgsea and msigdb libraries, and plotted using the ggplot2 and pheatmap packages.

### Assessment of cPLA2 Activity

Following the experimental interventions, cells were harvested and subjected to centrifugation at 2000 × g for 10 min at 4 °C. The obtained cell pellets were homogenized in 1 ml of ice‐cold extraction buffer (50 mm HEPES, pH 7.4, with 1 mm EDTA) per sample. Homogenates were subsequently centrifuged at 10 000 × g for 15 min at 4 °C, and the supernatants were collected for enzymatic analysis. Cytosolic phospholipase A2 (cPLA2) activity was quantified using the Cytosolic Phospholipase A2 assay Kit (Abcam; ab133090) following the instructions of the reagent supplier.

### Untargeted Lipidomic Analysis

For lipidomic analysis, cell samples were placed in 2 mL microcentrifuge tubes containing 100 mg glass beads and subjected to lipid extraction using 750 µL chloroform: methanol (2:1, v/v) with freeze‐thaw cycling and high‐frequency vortex mixing, followed by phase separation and organic layer collection. Extracts underwent secondary washing with chloroform: methanol, were concentrated to dryness under vacuum, reconstituted in 200 µL isopropanol, and filtered through 0.22 µm membranes. Chromatographic separation was performed on a Vanquish UHPLC system equipped with an XBridge Premier BEH C18 column (2.5 µm, 2.1 × 100 mm, 40 °C) with gradient elution using acetonitrile: water and isopropanol: acetonitrile mobile phases containing 10 mm ammonium formate. Mass spectrometry analysis was conducted using an Orbitrap Exploris 120 MS (Thermo Fisher Scientific, USA) with electrospray ionization source operating in Full MS‐ddMS2 mode, employing spray voltages of ±3.50/‐2.40 kV, capillary temperature of 325 °C, MS1 resolution of 60 000 (m/z 100–1000), MS/MS resolution of 30 000 (4 scans per cycle), and stepped collision energies of 15/25/30/40%. Raw mass spectrometry data underwent lipid peak annotation, alignment, and filtering using LipidSearch software (version 4.2.28) (35) with retention time tolerance of 0.25 and mass spectral score threshold of 3, followed by sum peak normalization for dataset consistency and statistical analysis using R software (version 4.4.1).

### Patient‐ and Mouse‐Derived Organoids

For patient‐derived organoids, freshly excised colorectal carcinoma tissues were mechanically minced, rinsed with ice‐cold PBS, and subjected to digestion with EDTA. Single‐cell suspensions were embedded in Matrigel and polymerized (37 °C, 30–60 min). Organoids were cultured in advanced DMEM/F12 supplemented with penicillin/streptomycin (1%), 2 mm GlutaMAX (Invitrogen), 10 mm HEPES (Invitrogen), 100 µg mL^−1^ primocin (InvivoGen), 1×B27 (Invitrogen), 1×N2 (Invitrogen), 1 mm N‐acetylcysteine (Sigma–Aldrich), and 10 nm gastrin I (Sigma–Aldrich), 50 ng mL^−1^ recombinant EGF (Invitrogen), 100 ng mL^−1^ FGF 10 (Invitrogen), 100 ng mL^−1^ Wnt3a (R&D), 2 µm A83‐01 (Sigma–Aldrich), 100 ng mL^−1^ recombinant Noggin (R&D), 10 µm Y27632 (Selleckchem), 1 µg mL^−1^ R‐spondin 1 (R&D), 10 mm nicotinamide (Sigma–Aldrich), and 10 µm SB202190 (Sigma). Established organoids were randomly allocated to control and treatment groups, with treatment cohorts exposed to TPhA (100 µm) alone or in combination with erastin (10 µm).

Mouse‐derived organoids were generated from tumor tissues obtained from wild‐type AOM‐DSS mice and PIR conditional knockout mice using an established protocol. The organoids underwent randomized treatment with 10 µm erastin or 5 µm AACOCF3, administered with or without 5 µm lipro‐1. Organoid morphology was documented via microscopy. All CRC samples were procured following the acquisition of written informed consent from the participating patients.

### Statistics

The statistical analyses were performed using GraphPad Prism software (version 8.0, San Diego, CA, USA) and R (version 4.4.1). Biological replication numbers for individual experiments were specified within corresponding figure legends. Unless otherwise noted, all data were expressed as mean ± standard deviation (SD) and represent results from a minimum of three independent biological replicates. Statistical significance comparisons were evaluated using two‐tailed unpaired Student's *t*‐test, 1‐way ANOVA with Dunnett's test, or 2‐way ANOVA with multiple comparisons. Statistical significance levels were represented as follows: ^*^ *P* < 0.05, ^**^ *P* < 0.01, ^***^ *P* < 0.001, ^****^ *P* < 0.0001; ns indicates non‐significance.

### Ethics Approval and Patient Consent Statement

This study obtained ethics approval for the collection and use of tissue microarrays (TMAs) and fresh samples from colorectal cancer (CRC) patients. Approval for TMAs was granted by the Human Ethics Committee of the IMCB, A*STAR (IRB: 2021–198), and the Singhealth Institutional Review Board, Singapore (2018‐2795). Patients undergoing primary surgical resection for colorectal cancer at Singapore General Hospital (SGH) provided written informed consent for research tissue donation. Fresh sample collection was approved by the Human Ethics Committee of the IMCB, A*STAR (IRB: 2022–094), and the National Healthcare Group Domain Specific Review Board (NHG DSRB Ref: 2022/00264). Patient samples were collected from the National University Hospital (NUH) following written informed consent obtained prior to surgery. All animal experimental procedures were conducted under approved IACUC protocols (ID 221 680 and ID 241 840).

## Conflict of Interest

The authors declare no conflict of interest.

## Author Contributions

W.S. provided funding, designed and performed the experiments, analyzed the data, drafted and revised the manuscript. Y.Q.O. conducted in vitro experiments, assisted in vivo experiments, and collected samples from patients. P.M. reviewed and gave suggestions on the revision of the manuscript. E.S.M.W. performed xenograft mouse models. A.M.V.V. and K.F. performed bioinformatics analysis for RNA‐seq results and the TCGA dataset. P.K.H.C. provided intellectual inputs, helped in data analysis, provided funding, polishing, and proofread the manuscript during the revision stage. W.L.T. provided advice for the design of the study. K. K. T. provided fresh CRC patients’ samples. I.B.T. provided PDCs, TMAs, and advice for the design of the study. V.T. contributed to conceptualization, experimental design, supervision, funding acquision, resource provision, manuscript review, and editing.

## Supporting information



Supporting Information

## Data Availability

The data that support the findings of this study are available from the corresponding author upon reasonable request.
