# Supplementary material for: Pirin Transcriptionally Regulates PLA2G4A To Inhibit Ferroptosis in Colorectal Cancer via Lipid Profile Remodeling
Source: Adv Sci (Weinh). 2025 Dec 16;13(11):e16385. doi: 10.1002/advs.202516385 (PMC12931218; doi:10.1002/advs.202516385)
Supplement: Supplementary file 1 — Supporting Information [file ADVS-13-e16385-s001.docx]

Supporting Information

**Pirin Transcriptionally Regulates PLA2G4A To Inhibit Ferroptosis In Colorectal Cancer Via Lipid Profile Remodeling**

*Wei Shi*, Yue Qi* *Ong, Prativa Majee, Esther SM Wong, Amhed Missael Vargas Velazquez, Kerem Fidan, Pierce Kah-Hoe Chow, Wai Leong Tam, Ker Kan Tan, Iain Beehuat Tan, Vinay Tergaonkar**

**Supplemental Methods and Materials**

*Reagents and antibodies*

Small molecule inhibitors—erastin (S7242), ferrostatin-1 (Fer-1, S7243), liproxstatin-1 (Lipro-1, S7699), RSL3 (S8155), deferoxamine mesylate (DFO, S5742), 3-methyladenine (3-MA, S2767), Z-VAD-FMK (S7023), and necrosulfonamide (Nec-1, S8251)—were obtained from Selleck Chemicals (Houston, TX, USA). AACOCF3 (HY-108611), tert-butylhydroquinone (TBHQ, HY-100489), imidazole ketone erastin (IKE, HY-114481), and Lipro-1 (HY-12726) were acquired from MedChemExpress (Monmouth Junction, NJ, USA). TPhA (ab144376) was bought from Abcam. FerroOrange (F374, Dojindo Laboratories, Japan) was employed for Fe²^+^ visualization in live-cell fluorescence imaging. Intracellular ROS detection utilized the BODIPY™ 581/591 C11 probe (D3861, Invitrogen, USA). Azoxymethane (AOM; A5486-25MG) and dextran sulfate sodium (DSS; molecular weight 36,000–50,000 Da) were procured from Sigma-Aldrich (St. Louis, MO, USA) and MP Biomedicals (Solon, OH, USA), respectively, for colitis-associated cancer modelling.

Primary antibodies included anti-PIR (ab227280; rabbit monoclonal) and anti-NRF2 (ab137550; rabbit monoclonal) from Abcam (Cambridge, UK). Anti-cPLA2 (#5249; rabbit monoclonal) was sourced from Cell Signaling Technology (Danvers, MA, USA). The PLA2G4A antibody (28924-1-AP; rabbit polyclonal), Ki67 antibody (27309-1-AP; rabbit polyclonal), and 4-HNE (ab46545; 1:200 dilution; mouse monoclonal) for immunohistochemistry were purchased from Proteintech (Rosemont, IL, USA) and Abcam, respectively.

*Bioinformatics analysis*

Normalized RNA counts (TPM) from tumorous Colon Adenocarcinoma samples belonging to the TCGA cohort patients (COADREAD, https://www.cancer.gov/tcga) and associated metadata were downloaded from Xena browser via the UCSCXenaTools^1^ library in R. The 383 samples displaying various levels of pirin (PIR) expression were grouped based on their quartile location within the PIR expression distribution. The lowest (<= 7.099 tpm, 96 samples) and the highest (>= 8.262 tpm, 96 samples) quartiles were selected for further comparison. CSimilarly, differential gene expression (DGE) analysis between the two groups was performed using DeSEQ2.^2^ Only significantly differentially expressed genes, p value < 0.05 and log2FC of absolute 1, were selected for gene set enrichment analysis (GSEA). GSEA analysis on a set of human canonical pathways (CP2) was performed using the fgsea^3^ library in R, and among the significant pathways, the “Ferroptosis” WikiPathway was selected for illustrative purposes.

*Cell culture*

The American Type Culture Collection (ATCC) provided human CRC cell lines (HCT15, HCT116, DLD1, LoVo, SW480, RKO, Caco2 and Colo205), murine CRC cell line (CT26), fibrosarcoma cell line (HT1080) and HEK293T cells. All cell lines were cultured at 37°C in a humidified incubator in the presence of 5% CO2 (volume/volume; v/v). HCT15, CT26, and HEK293T were maintained in Dulbecco’s Modified Eagle Medium (DMEM) with 10% (volume/volume; v/v) fetal bovine serum (FBS) (Gibco) and 1% (v/v) penicillin/streptomycin. HT1080 cells were cultured in Eagle’s Minimum Essential Medium (EMEM) containing 10% FBS. Primary CRC cell lines were maintained in Matrigel-coated 6-well tissue culture-treated plates (Falcon) precoated with the Coating Matrix Kit (Gibco). The cells were cultured in DMEM/F12 medium (Gibco) supplemented with penicillin-streptomycin (Gibco), B27 (without vitamin A, Gibco), 20 ng/ml epidermal growth factor (Gibco), and 10 ng/ml basic fibroblast growth factor (bFGF; Gibco). Cell lines were routinely screened for mycoplasma contamination using the Mycoplasma PCR Detection Kit (No. G238, abm) and were regularly validated to confirm purity and freedom from infection.

*CRISPR/Cas9-associated plasmids construction*

CRISPR/Cas9 technology was employed to perform the targeted knockout of PIR. Single guide RNAs (sgRNAs) were designed using an online tool (http://crispr.mit.edu/) to identify the most efficient target site for PIR. A pair of guide RNA spacer sequences was selected and subsequently cloned into pSpCas9(BB)-2A-GFP (PX458) (Addgene, plasmid #48138) plasmids according to the standard cloning protocol established by the Zhang lab. The sgRNAs were listed in Table S1. The plasmids were transfected into cells (HCT15, HCT116, HT1080, or CT26) using Lipofectamine LTX reagent (Thermo Fisher Scientific). Following transfection, cells were sorted into 96-well plates for screening via flow cytometry. Knockout efficiency was confirmed by immunoblotting, and single-cell clones with successful knockouts were selected and expanded for subsequent analysis. The sequences of the gRNA were listed in Table S1.

*Plasmids*

All plasmids utilized in this study (except viral packaging plasmids or those explicitly specified by suppliers) were constructed in the VT lab. HEK293T cells were used for lentiviral packaging and transduction. Using the pLenti vector backbone, Flag-PIR and no-tag PLA2G4A were cloned into the pLV-EF1α-IRES-Blast vector (Addgene, plasmid #85133).

For knockdown experiments, three hairpin shRNAs targeting the coding sequences (CDS) of PIR, NRF2, and PLA2G4A were designed and inserted into the pLKO.1-puro vector (Addgene, plasmid #8453). The targeting sequences are shown in Table S1. A non-targeting control shRNA was labelled as shNC. High-titer lentiviral particles were generated in 293T cells, and virus-containing medium was collected 48 hours post-transfection. Infected cells were selected by puromycin (2 μg/ml), and knockdown efficiency was evaluated at the mRNA and protein levels. The primers for cloning were presented in Table S1.

*CCK-8 assay*

Cell viability was assessed using the Cell Counting Kit-8 (CCK8; DOJD/CK04; Dojindo Laboratories). In brief, cells were plated in 96-well plates and cultured with the indicated drugs the following day. At the designated time points, the fresh medium with 10% CCK8 replaced the old medium. After incubation at 37°C for 2 hours, the absorbance at 450 nm was measured using a microplate reader. The absorbance was used to determine cell viability, expressed as a relative percentage, with 100% corresponding to the untreated control.

*RNA isolation, reverse transcription, and qPCR*

Total RNA was isolated from cultured cells or tissues using TRIzol reagent (Thermo Fisher Scientific), followed by purification with Nucleospin RNA columns (Macherey-Nagel) according to the manufacturer’s specifications. Complementary DNA (cDNA) synthesis was performed using 1 μg of total RNA with the SuperScript VILO cDNA Synthesis kit (11754050; Thermo Fisher Scientific). Quantitative PCR (qPCR) analyses were performed using the CFX96 Real-Time System (Bio-Rad) with SsoAdvanced Universal SYBR Green Supermix (Bio-Rad). Relative gene expression levels were determined using the comparative 2−ΔΔCt method, employing β-actin and GAPDH as the endogenous reference gene and normalization performed relative to a designated control sample within each experimental cohort. Primer sequences and specifications are detailed in Table S1.

*Agarose gel electrophoresis*

Agarose gels (2% w/v) were prepared in 1× Tris-borate-acid-EDTA (TBE) buffer containing FloroSafe DNA stain (BIO-5170, 1st BASE, Asia). DNA samples underwent electrophoretic separation at 120 V for 25-35 minutes in 1× TBE buffer. Post-electrophoretic visualization and documentation were performed using a ChemiDoc™ imaging system (Bio-Rad, USA). DNA fragments were detected under ultraviolet illumination, and molecular weight determination was facilitated using ExactMark™ 1 kb DNA Ladder (1st BASE) as the size standard. The primers for genotyping were shown in Table S1.

*Western blot*

Cellular lysis was performed in Totex lysis buffer composed of 20 mM HEPES (pH 7.9), 150 mM NaCl, 20% glycerol, 1% NP-40, 1 mM MgCl2, 0.5 mM EDTA, and protease inhibitor cocktail (Roche Applied Science) with incubation for 30 min at 4 °C. Cell lysates were subsequently sonicated for 5 minutes using a Bioruptor UCD-200 (Diagenode). Following centrifugation (15, 000g for 15 min, 4°C), supernatants containing soluble proteins were collected. Protein concentrations were determined using the Bradford protein assay (Bio-Rad). Equivalent protein quantities were resolved by electrophoresis on 4-12% Bis-Tris polyacrylamide gels (NuPAGE, Thermo Fisher Scientific). Resolved proteins were electrophoretically transferred to a methanol-activated polyvinylidene difluoride (PVDF) membranes (Bio-Rad) and subsequently blocked with 5% non-fat milk for 2 hours at room temperature, followed by overnight incubation with appropriate primary antibodies at 4°C and subsequent incubation with the species-matched horseradish peroxidase (HRP)-conjugated secondary antibodies for 1 h at room temperature. Immunoblotting was visualized using the ClarityTM Western ECL Substrate (Bio-Rad) and chemiluminescent detection.

*Immunohistochemistry*

Tissue microarrays (TMAs) were subjected to hematoxylin and eosin (H&E) staining to confirm representative tumor regions. Immunohistochemical (IHC) analysis was performed on paraffin-embedded sections from patient samples or xenograft tumor tissues. Tissue sections underwent deparaffinization in xylene followed by rehydration through a graded ethanol series. Endogenous peroxidase activity was quenched by treatment with methanol/H₂O₂ solution for 15 minutes. Antigen retrieval was carried out for 45 minutes in citrate buffer heated to 95°C. Sections were subsequently blocked for 1 hour at room temperature, followed by overnight incubation at 4°C with primary antibodies: anti-PIR (1:300, ab227280, Abcam), anti-PLA2G4A (1:300, 68133-1-Ig, Proteintech), anti-4-HNE (1:300, ab46545, Abcam), and anti-Ki67 (1:300, 27309-1-AP, Proteintech). After rinsing, sections were treated with appropriate HRP-conjugated secondary antibodies for 1 hour at room temperature. Chromogenic detection was achieved using 3,3'-diaminobenzidine (DAB). After dehydration through graded ethanol and xylene, the sections were mounted with neutral resin. Immunohistochemical scoring was performed using a semi-quantitative assessment system. Staining intensity was graded: 0 (negative), 1 (weak), 2 (moderate), 3 (strong). The percentage of immunopositive cells was categorized as follows: 0 (0%), 1+ (1–25%), 2+ (26–50%), 3+ (51–75%), 4+ (>75%). A staining index (SI) was calculated as the product of the intensity score and the positive cell proportion category (SI = Intensity × Percentage Category). The median SI value across all samples served as the threshold to define high versus low expression groups for PIR and PLA2G4A. Immunohistochemical evaluation was conducted independently by two pathologists who were blinded to the experimental conditions.

*Xenograft mouse models*

To confirm the impact of PIR or AACOCF3 in ferroptosis in vivo, wild-type CT26 or PIR-knockout cells (1 × 10^6^ cells per injection site) were subcutaneously injected into the right dorsal region of 6- to 8-week-old immunocompetent BALB/c mice (InVivos). Upon tumor establishment (volume 50-70 mm³ at day 7), mice were randomly allocated to control or treatment cohorts. Treatment groups received imidazole ketone erastin (IKE, 30 mg/kg, intraperitoneal, every other day) or AACOCF3 (10 mg/kg, intraperitoneal, every other day) either alone or in combination with Liproxstatin-1 (10 mg/kg, intraperitoneal, every other day) commencing at day 7 for a 2-week duration. Control animals received vehicle solution [10% DMSO, 40% PEG 300 (Sigma Aldrich, Cat #90878), and 5% Tween-80 (Sigma Aldrich, Cat #P4780) in saline]. Tumor dimensions were measured biweekly using calipers, and volumes were calculated as (length × width^2^)/2. The mice were sacrificed when their tumors reached approximately 15 mm in diameter, and subsequently, the tumors were dissected for weighing and other quantifications.

*AOM-DSS mouse models*

For azoxymethane (AOM) and dextran sulfate sodium (DSS)-induced colorectal carcinogenesis, 8–10-week-old *PIR^+/+^*; *Villin-Cre^ERT2^* and *PIR^fl/fl^*; *Villin-Cre^ERT2^* mice received a single intraperitoneal injection of AOM (A5486, Sigma-Aldrich) at a dose of 10 mg/kg. This was followed by three cycles of 1.5% DSS (160110, MP Biomedicals) administration via drinking water for 7 consecutive days, alternating with 14-day intervals of standard water. At the conclusion of the DSS treatment, *PIR^+/+^*; *Villin-Cre^ERT2^* and *PIR^fl/fl^*; *Villin-Cre^ERT2^* mice were randomized into control or treatment cohorts. Therapeutic interventions consisted of Lipro-1 (10 mg/kg) or AACOCF3 (10 mg/kg) administered intraperitoneally every other day. Distal colon tissues were harvested for quantitative assessment of tumor multiplicity and volume. Total polyp count represented the cumulative number of polyps per animal, while the polyp burden was quantified as the cumulative diameter of all polyps observed.

**
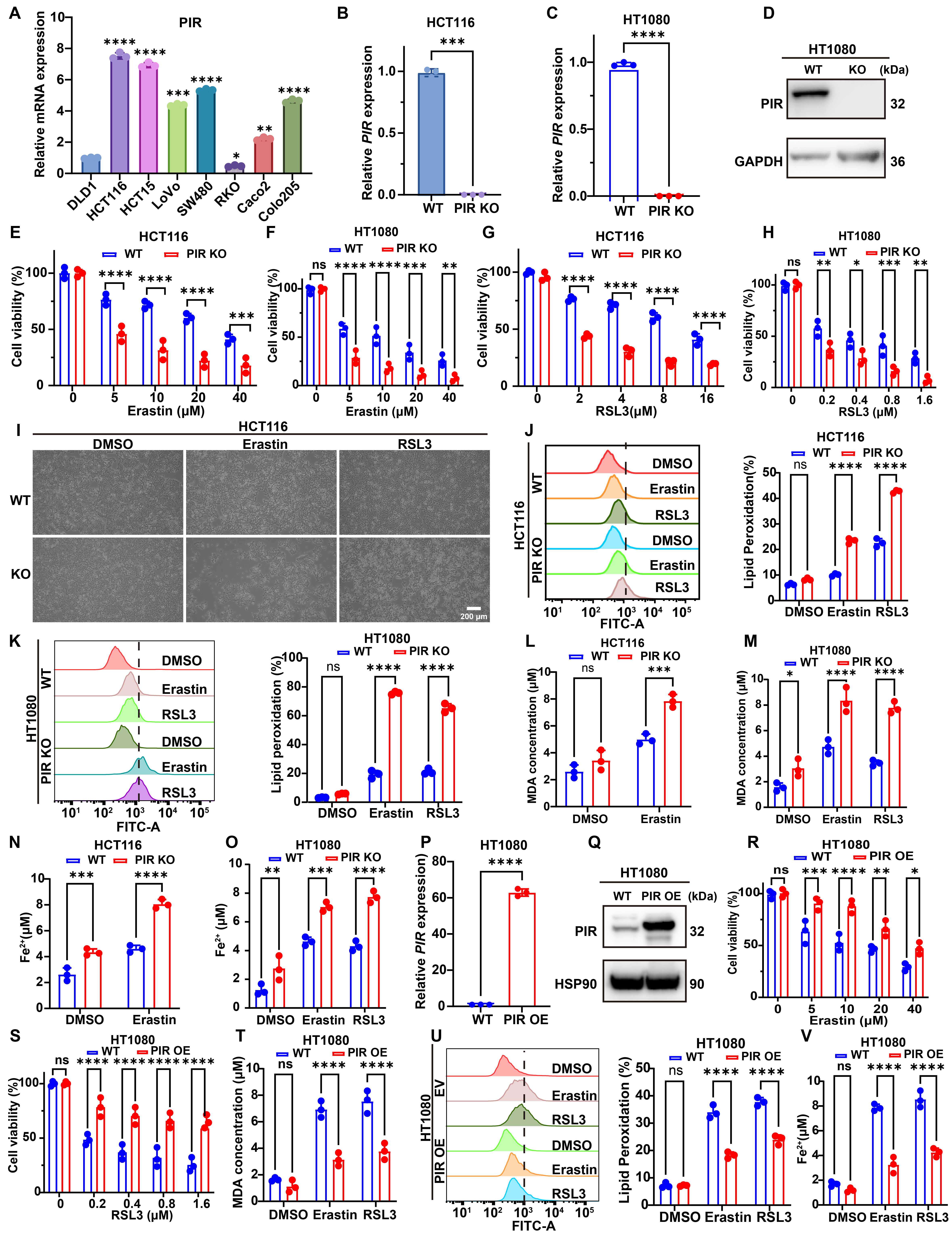
Figure S1.** **PIR ablation enhances ferroptosis inducer cytotoxicity in HCT116 and HT1080 cells.** A) qPCR of *PIR* mRNA expression across CRC cell lines. B, C) *PIR* mRNA levels in WT and PIR-KO HCT116 (B) and HT1080 (C) cells. D) Immunoblot analysis confirming PIR deletion in HT1080 cells. E, F) Dose-response cell viability in PIR-KO versus WT cells treated with erastin in HCT116 (E) and HT1080 cells (F). G, H) Dose-dependent cell viability curves comparing PIR-KO and WT cells following RSL3 treatment in HCT116 (G) and HT1080 (H) cells. I) Representative phase-contrast micrographs depicting ferroptosis-associated morphological alterations in erastin- and RSL3-treated PIR-depleted HCT116 cells. Scale bar: 200 μm. J, K) C11-BODIPY fluorescence analysis indicating increased lipid peroxidation in PIR-KO HCT116 (J) and HT1080 cells (K). L, M) MDA accumulation in PIR-KO cells under erastin administration in HCT116 (L) and HT1080 cells (M). N, O) Intracellular Fe²⁺ levels in PIR-KO HCT116 (N) and HT1080 (O) cells after erastin or RSL3 treatment. P, Q) Validation of PIR OE by qRT-PCR (P) and Western blot (Q) analyses. R, S) Cell viability assays in PIR-OE HT1080 cells treated with erastin (R, 12 h) or RSL3 (S, 2 h). T, U, V) MDA levels (T), lipid peroxidation (U), and intracellular Fe^2+^ content (V) in WT and PIR-OE cells following erastin (5 μM) or RSL3 (200 nM) treatment. Data are shown as mean ± SD from three independent experiments. ^*^*P*<0.05, ^**^*P*<0.01, ^***^*P*<0.001, ^****^*P*<0.0001 by 1-way ANOVA (A), 2-tailed unpaired Student’s *t*-test (B, C and P) and 2-way ANOVA with multiple comparisons (E-H, J-O, and R-V).

**
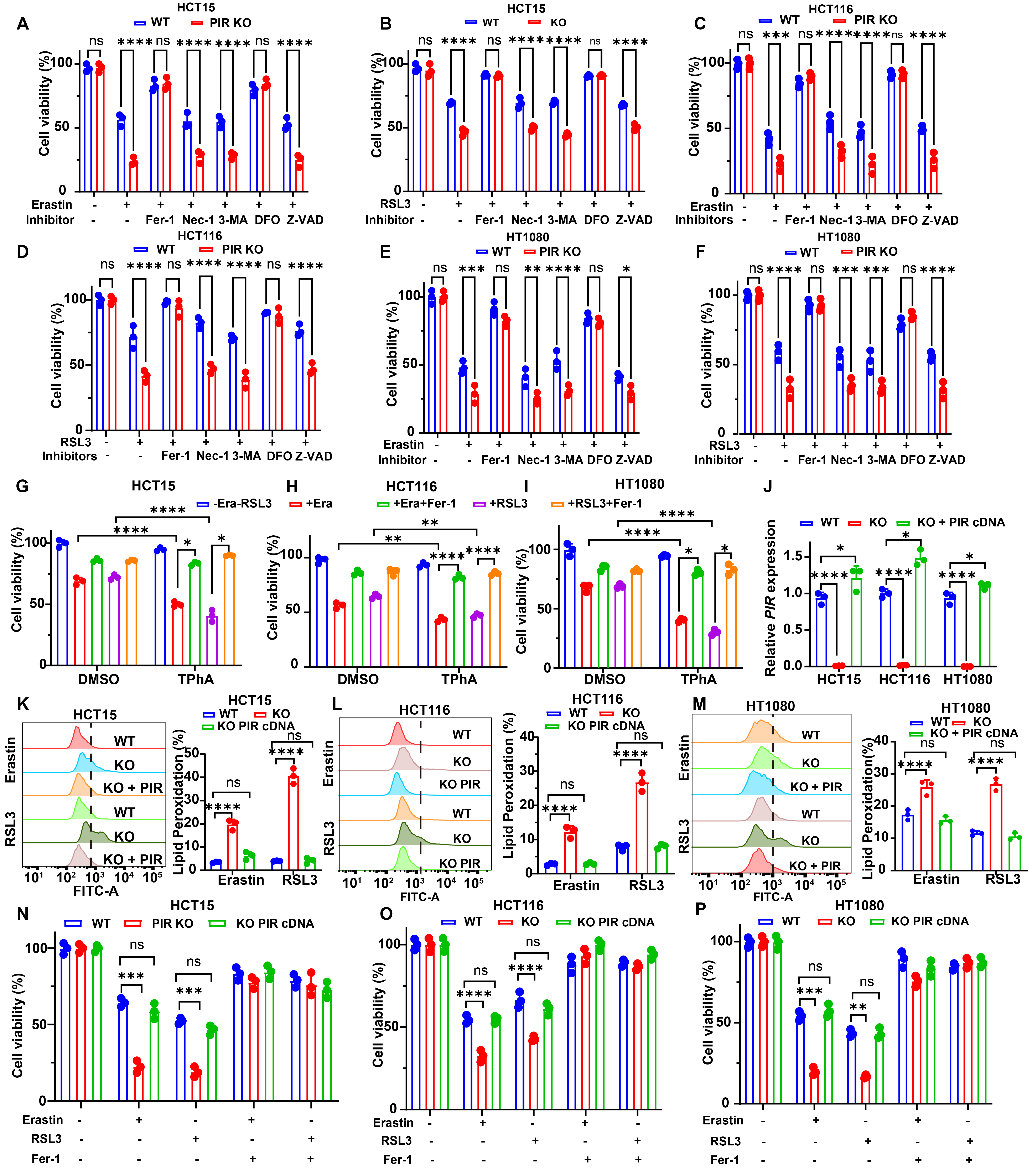
**

**Figure S2.** **PIR deletion and pharmacological inhibition synergize with ferroptosis inducers (FINs)**. A, B) Viability of WT and PIR-KO HCT15 cells treated with erastin (10 μM; A) or RSL3 (4 μM; B) in the presence or absence of ferrostatin-1 (Fer-1, 2 μM), deferoxamine (DFO, 5 μM), necrostatin-1 (Nec-1, 10 μM), 3-methyladenine (3-MA, 100 μM), or Z-VAD-FMK (10 μM). C, D) Viability of WT and PIR-KO HCT116 cells treated with erastin (10 μM; A) or RSL3 (4 μM; B) combined with the same panel of cell death inhibitors. E-F) Viability of WT and PIR-KO HT1080 cells treated with erastin (5 μM; E) or RSL3 (200 nM; F) with or without the inhibitors. G-I) Cell viability of HCT15 (G), HCT116 (H) or HT1080 (I) cells treated with erastin or RSL3 in the absence or presence of TPhA (50 μM). J) qPCR validation of PIR expression in WT, PIR-KO, and PIR-reconstituted HCT15, HCT116 and HT1080 cells. K-M) PIR reconstitution rescues erastin- or RSL3-induced lipid peroxidation in PIR-deleted HCT15 (K), HCT116 (L), and HT1080 (M) cells. N-P) CCK-8 cell viability assays in PIR-reconstituted HCT15 (N), HCT116 (O), and HT1080 (P) cells treated with erastin or RSL3 in the absence or presence of Fer-1. Data represent mean ± SD from three independent experiments. ^*^*P*<0.05, ^**^*P*<0.01, ^***^*P*<0.001, ^****^*P*<0.0001, by 2-way ANOVA with multiple comparisons (A-F, D-I and K-P), by 1-way ANOVA with Dunnett’s test (J).

**
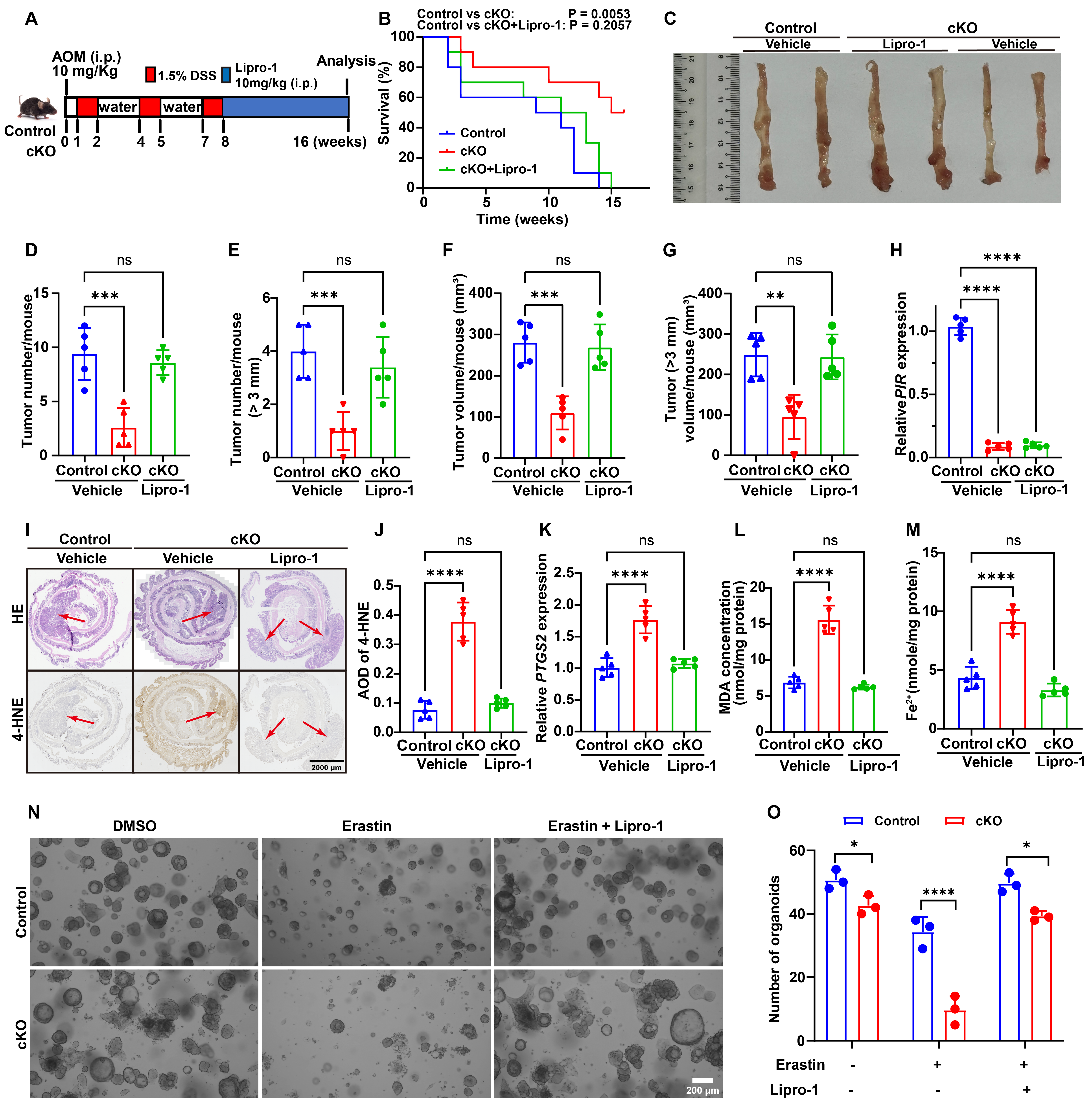
Figure S3. Ferroptosis inhibition abrogates the suppressive effect of PIR deficiency on AOM/DSS-induced colorectal carcinogenesis.** A) Experimental design for AOM/DSS-induced tumorigenesis in control and PIR cKO mice with Lipro-1 (10 mg/kg, i.p.) or vehicle. B) Kaplan-Meier survival curves for control and cKO mice following AOM/DSS ± Lipro-1 (n = 10/group). C) Representative macroscopic images of tumor-bearing colorectum from each AOM/DSS-treated cohort. D, E) Quantification of total tumor number (D) and the number of tumors >3 mm (E) per mouse (n = 5/group). F, G) Assessment of total tumor burden (F) and the volume of tumors greater than 3 mm (G) per mouse across treatment groups (n = 5 per group). H) qRT-PCR analysis of *PIR* expression in excised colonic tumors. I) Representative images of H&E and 4-HNE immunohistochemical staining in colonic tissues following Lipro-1 treatment. Scale bar: 2000 μm. J) Quantification of 4-HNE staining intensity by AOD (n = 5/group). K) qRT-PCR analysis of *PTGS2* expression in colonic tumors. L, M) Measurement of MDA (L) and Fe^2+^ (M) levels in excised tumors (n = 5/group). N) Representative images of tumor-derived organoids from control and cKO mice treated with vehicle or erastin in the absence or presence of Liproxstatin-1. Scale bar: 200 μm. O) Quantification of organoids corresponding to conditions in (N). Data represent mean ± SD. ^*^*P*<0.05, ^**^*P*<0.01, ^***^*P*<0.001, ^****^*P*<0.0001, determined by 1-way ANOVA with Dunnett’s test (D-H and J-M) and 2-way ANOVA with multiple comparisons (O).

**
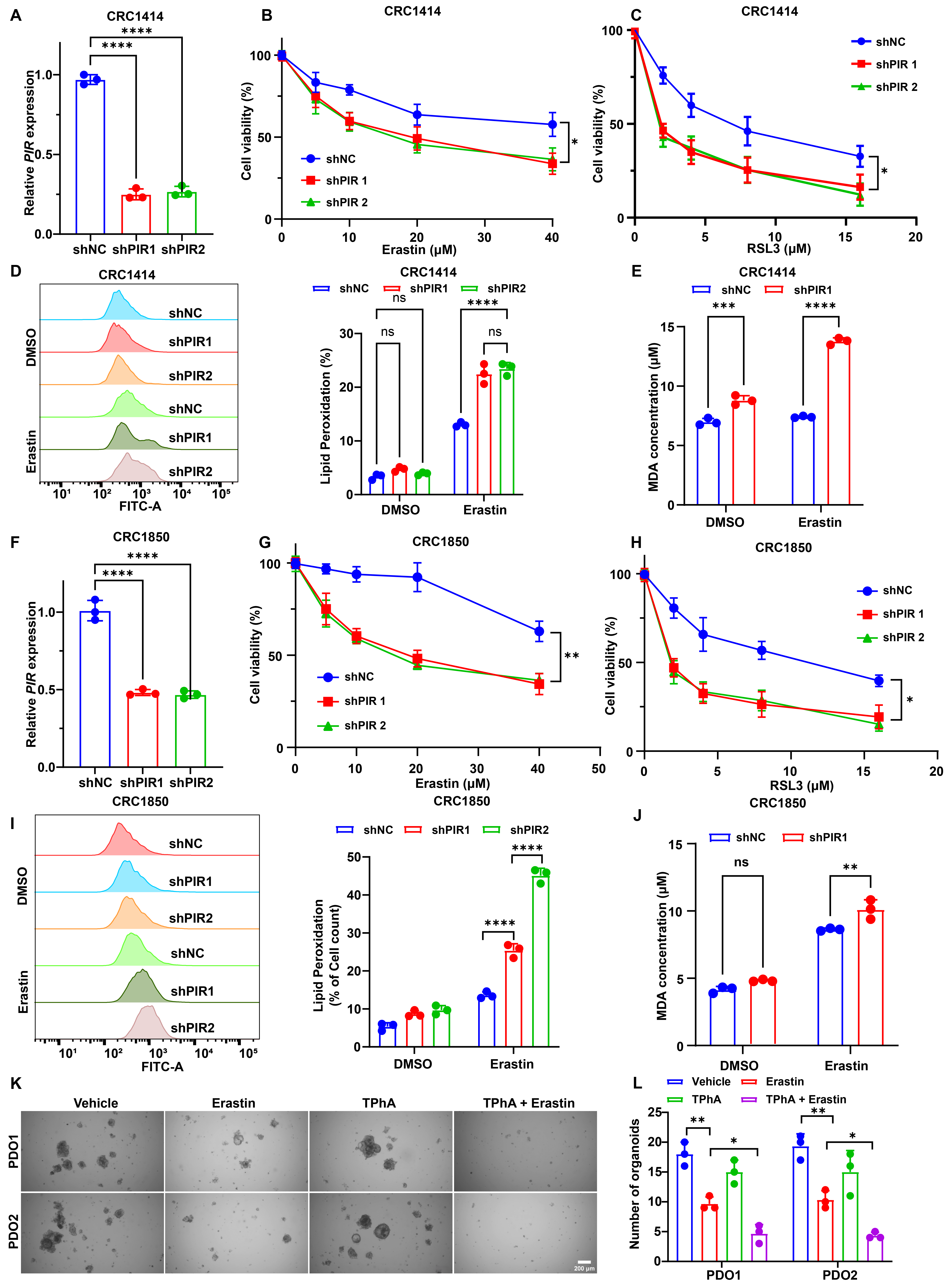
**

**Figure S4. PIR deficiency enhances ferroptotic sensitivity in patient-derived cells and organoids.** A) qPCR analysis of *PIR* knockdown efficiency in CRC1414 cells. B, C) PIR knockdown enhanced CRC1414 cell sensitivity to erastin (B) and RSL3 (C). D) C11-BODIPY fluorescence indicating increased lipid peroxidation in PIR-depleted CRC1414 cells. E) MDA accumulation in PIR-depleted CRC1414 cells following erastin treatment. F-H) PIR knockdown validation in CRC1850 cells (F) with enhanced sensitivity to erastin (G) and RSL3 (H). I) Lipid peroxidation in PIR-depleted CRC1850 cells post-erastin treatment. J) MDA quantification in erastin-treated CRC1850 cells with PIR knockdown. K) PDOs treated with erastin ± TPhA. Scale bar: 200 μm. L) Organoid quantification for experimental groups in (K). Data represent mean ± SD from three independent experiments. ^*^*P*<0.05, ^**^*P*<0.01, ^***^*P*<0.001, ^****^*P*<0.0001, by 1-way ANOVA with Dunnett’s test (A and F) or 2-way ANOVA with multiple comparisons (B-E, G-J, L).


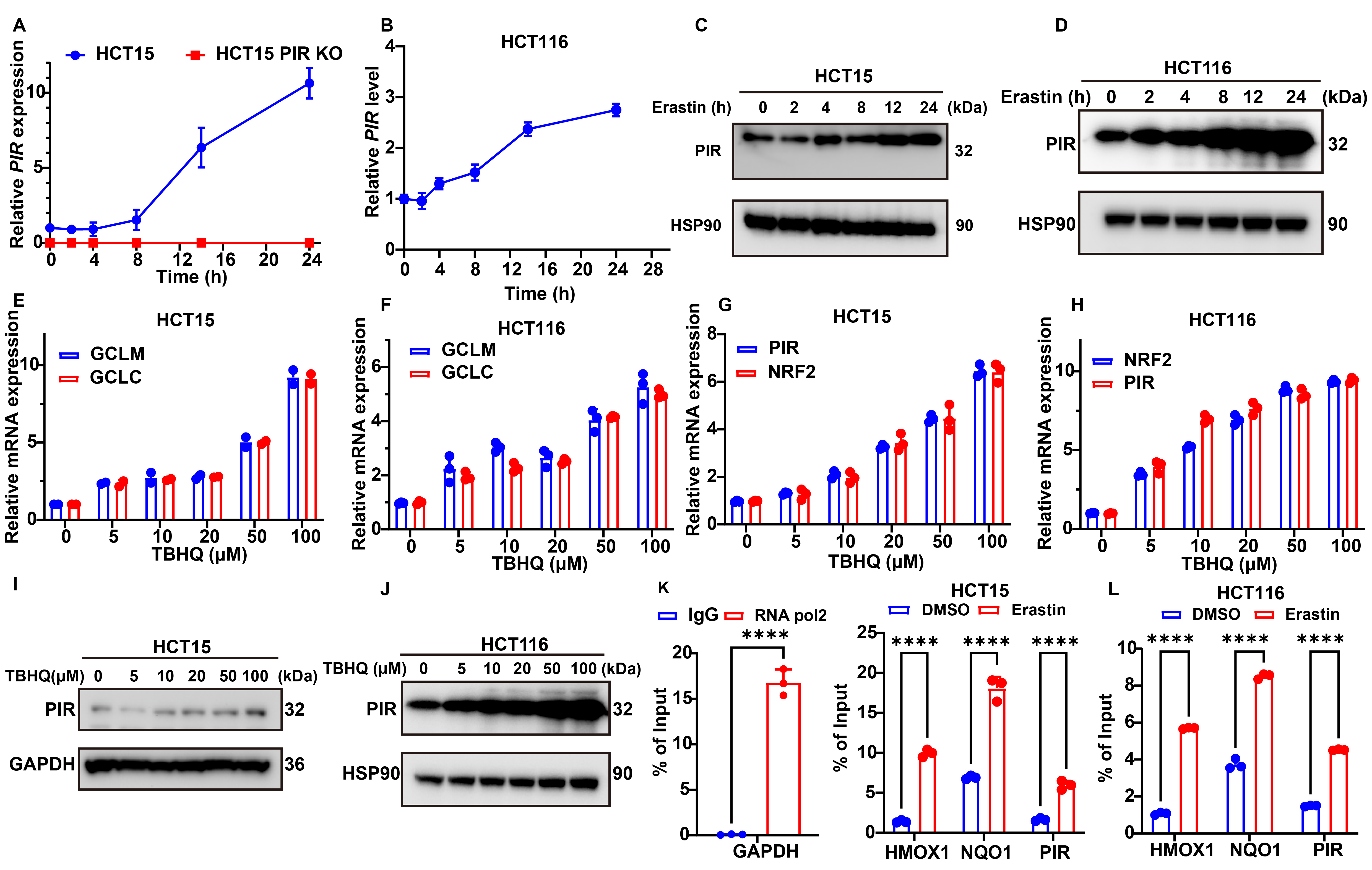


**Figure S5.** **PIR functions as an NRF2-regulated redox sensor modulating ferroptosis in CRC cells.** A, B) Time-course analysis of *PIR* mRNA expression in HCT15 (A) and HCT116 (B) cells following erastin (10 μM) treatment, as determined by qPCR. C, D) Immunoblot analysis demonstrating time-dependent PIR protein expression in HCT15 (C) and HCT116 (D) cells upon erastin (10 μM) treatment. E, F) Dose-dependent induction of *GCLM* and *GCLC* expression by TBHQ (0–100 μM, 24 h) in HCT15 (E) and HCT116 (F) cells. G, H) Quantitative PCR validation of TBHQ-mediated *PIR* upregulation in HCT15 (G) and HCT116 (H) cells. I, J) Western blot analysis confirming TBHQ-induced PIR protein upregulation in HCT15 (I) and HCT116 (J) cells. K, L) The successful implementation of ChIP-qPCR was validated by significant enrichment of RNA polymerase II (Pol2) at the GAPDH promoter. Subsequent analysis revealed a marked increase in NRF2 binding at the HMOX1, NQO1 and PIR promoter regions in erastin-treated (10 μM, 24 h) HCT15 and HCT116 cells compared to untreated controls. Data are presented as mean ± SD from three independent experiments. ^****^*P*<0.0001 by 2-tailed unpaired Student’s *t*-test (K) or 2-way ANOVA with multiple comparisons (K and L).

**
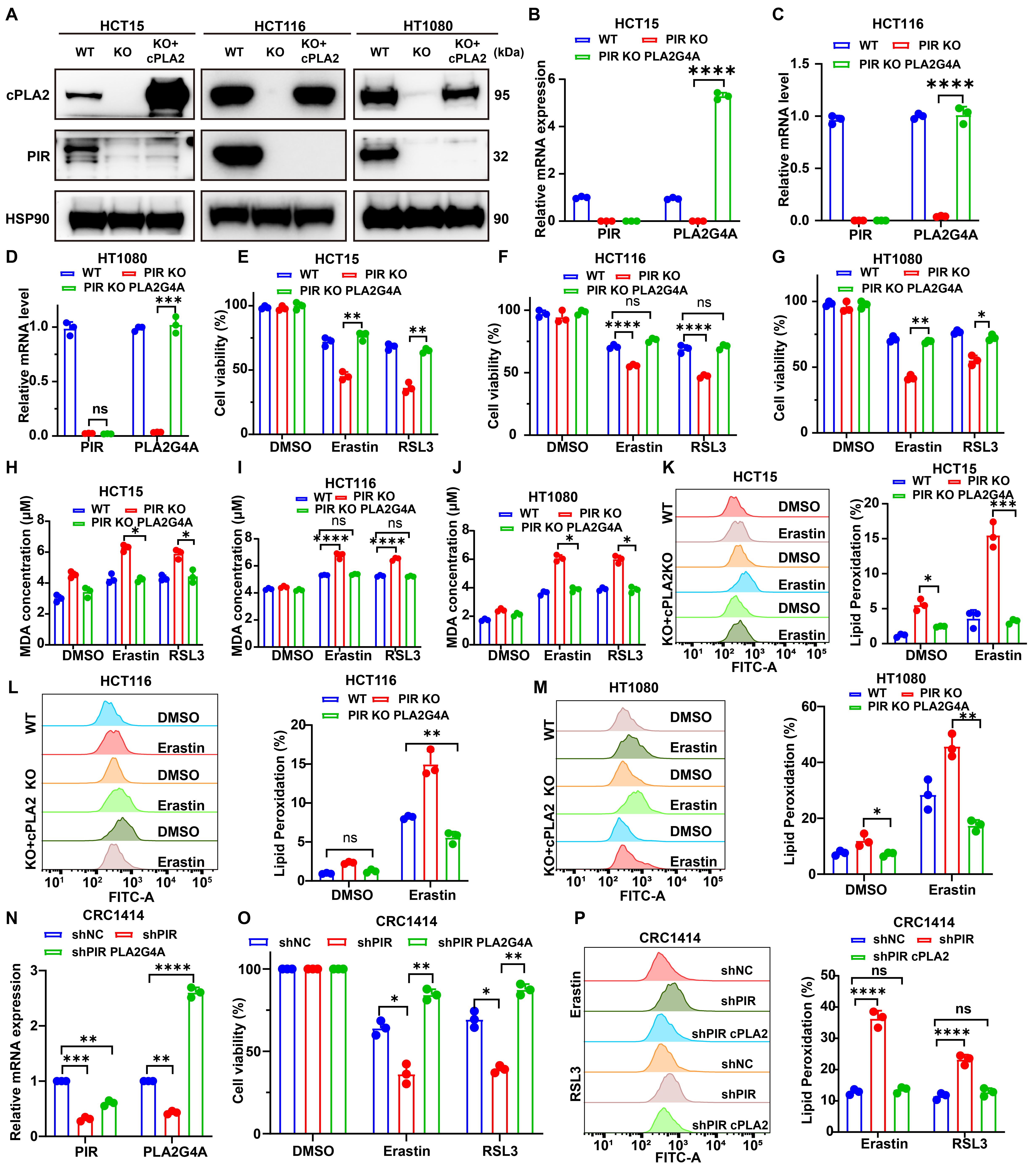
Figure S6. Reconstitution of PLA2G4A rescues ferroptosis resistance in PIR-deficient cells.** A) Immunoblot analysis demonstrating restoration of cPLA2 protein expression in PIR-KO HCT15, HCT116, and HT1080 cells. B-D) Validation of *PLA2G4A* restoration in PIR-KO HCT15 (B), HCT116 (C), and HT1080 (D) cells by qRT-PCR. E-G) CCK-8 viability assays demonstrating PLA2G4A-mediated protection against erastin/RSL3-induced death in PIR-KO HCT15 (E), HCT116 (F) and HT1080 (G) cells. H-J) Quantification of MDA levels in PLA2G4A-reconstituted PIR-KO HCT15 (H), HCT116 (I), and HT1080 (J) cells following erastin or RSL3 treatment. K-M) C11-BODIPY fluorescence analysis demonstrating that PLA2G4A restoration attenuates PIR deletion-enhanced lipid peroxidation in HCT15 (K), HCT116 (L), and HT1080 (M) cells. N) qRT-PCR confirmation of *PLA2G4A* re-expression in PIR-depleted CRC1414 cells. O, P) PLA2G4A restoration rescues cell viability (O) and reduces lipid peroxidation (P) in CRC1414 cells following erastin or RSL3 treatment. Data represent mean ± SD from three independent experiments. ^*^*P*<0.05, ^**^*P*<0.01, ^***^*P*<0.001, ^****^*P*<0.0001, by 2-way ANOVA with multiple comparisons (B-P).

**
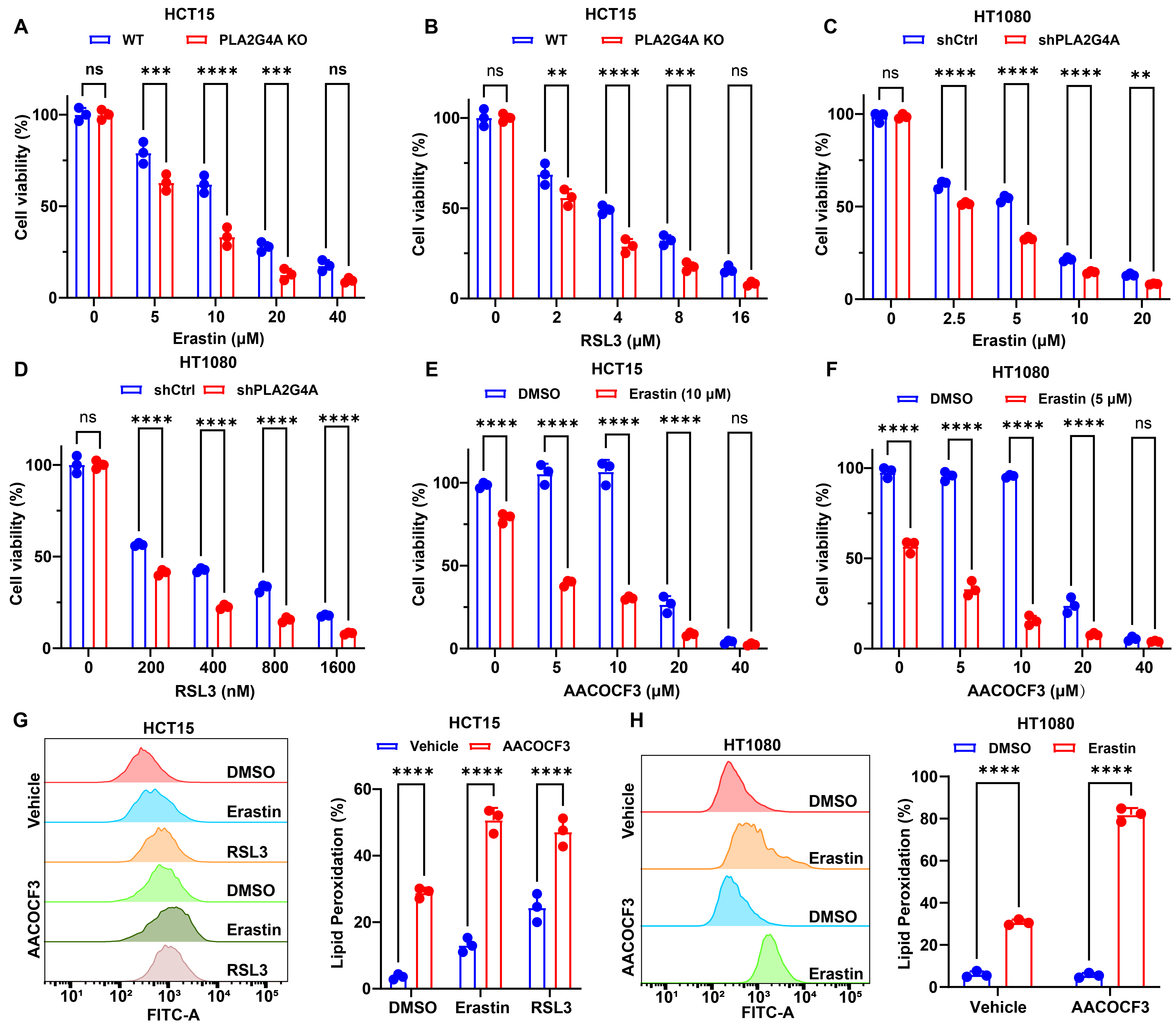
Figure S7. The PIR-PLA2G4A axis confers ferroptosis resistance in CRC cells.** A, B) CCK-8 cell viability assays in PLA2G4A-knockout HCT15 cells following erastin (A) or RSL3 (B) treatment. C, D) Enhanced ferroptosis sensitivity of PLA2G4A-knockdown HT1080 cells upon erastin (C) and RSL3 (D) treatment. E, F) AACOCF3 treatment potentiates erastin-induced cytotoxicity in HCT15 (E) and HT1080 (F) cells. G, H) C11-BODIPY fluorescence analysis demonstrating increased lipid peroxidation in HCT15 (G) and HT1080 (H) cells treated with erastin or RSL3 in the presence of AACOCF3. Data represent mean ± SD from three independent experiments. ^**^*P*<0.01, ^***^*P*<0.001, ^****^*P*<0.0001, by 2-way ANOVA with multiple comparisons (A-H).

**
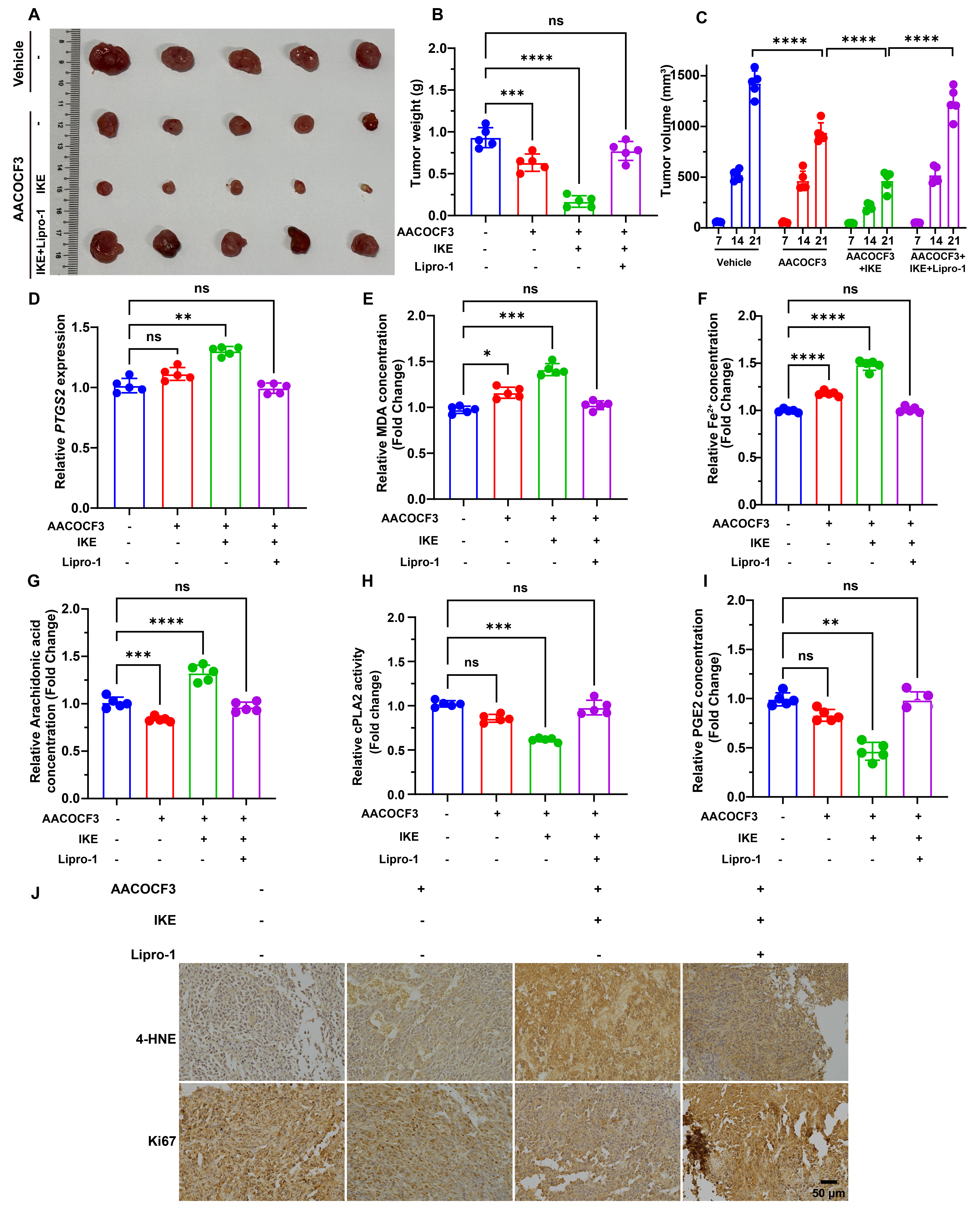
**

**Figure S8. Pharmacological inhibition of PLA2G4A synergizes with ferroptosis induction to suppress tumor growth *in vivo*.** A-C) CT26 cells were implanted subcutaneously in BALB/c mice, followed by pharmacological interventions (n = 5 per group). Representative images of excised tumors (A), tumor weights (B), and tumor volumes (C). D-I) Biochemical analysis of excised tumor tissues: *PTGS2* mRNA expression levels (D), MDA accumulation (E), Fe^2+^ concentrations (F), arachidonic acid levels (G), relative cPLA2 activity (H), and PGE2 quantification (I). J) Representative immunohistochemical staining for 4-HNE and Ki-67 in tumor xenografts. Scale bars: 50 μm. Data represent mean ± SD. ^*^*P*<0.05, ^**^*P*<0.01, ^***^*P*<0.001, ^****^*P*<0.0001, by 1-way ANOVA with Dunnett’s test (B and D-I) and 2-way ANOVA with multiple comparisons (C).

References

1. Wang S, Liu X. The UCSCXenaTools R package: a toolkit for accessing genomics data from UCSC Xena platform, from cancer multi-omics to single-cell RNA-seq. *Journal of Open Source Software* 2019, **4**(40)**:** 1627.

2. Love MI, Huber W, Anders S. Moderated estimation of fold change and dispersion for RNA-seq data with DESeq2. *Genome biology* 2014, **15**(12)**:** 550.

3. Korotkevich G, Sukhov V, Budin N, Shpak B, Artyomov MN, Sergushichev A. Fast gene set enrichment analysis. *biorxiv* 2016**:** 060012.

**Table S1. Sequences of all the primers used in this study.**

| **Names** | **Sequences (5’→3’)** |
| --- | --- |
| qhPIR F | AAGCCCTGGGAATAAAGTCCA |
| qhPIR R | AGCACTGCTGTGTGATGAGG |
| qhactin F | GCCAACCGCGAGAAGATGA |
| qhactin R | CCATCACGATGCCAGTGGTA |
| qhGAPDH F | GAGTCAACGGATTTGGTCGT |
| qhGAPDH R | GACAAGCTTCCCGTTCTCAG |
| qmPIR F | AAGTCGAAGGTTTACACTCGC |
| qmPIR R | AGCTTGTCCACCCTTTAGGAA |
| qmPTGS2 F | GGGAGTCTGGAACATTGTGAA |
| qmPTGS2 R | GTGCACATTGTAAGTAGGTGGACT |
| qhPLA2G4A F | TACCAGCACATTATAGTGGAGCA |
| qhPLA2G4A R | GCTGTCAGGGGTTGTAGAGAT |
| qhVIL F | CTGAGCGCCCAAGTCAAAG |
| qhVIL R | AGCAGTCACCATCGAAGAAGC |
| qhASCL2 F | AACTTGAGCTGCTGGAGGGACA |
| qhASCL2 R | TCTTGGCCAGCATGGAAAACTC |
| qhDAPK1 F | ACGTGGATGATTACTACGACACC |
| qhDAPK1 R | TGCTTTTCTCACGGCATTTCT |
| qhPTGS2 F | TCCCTTGGGTGTCAAAGGTAAA |
| qhPTGS2 R | TGGCCCTCGCTTATGATCTG |
| qhGCLM F | CATTTACAGCCTTACTGGGAGG |
| qhGCLM R | ATGCAGTCAAATCTGGTGGCA |
| qhGCLC F | GGCGATGAGGTGGAATACAT |
| qhGCLC R | GTCCTTTCCCCCTTCTCTTG |
| qhNFE2L2 F | CAACTACTCCCAGGTTGCCC |
| qhNFE2L2 R | AGTGACTGAAACGTAGCCGA |
| qhNFE2L2 F | CAACTACTCCCAGGTTGCCC |
| qhNFE2L2 R | AGTGACTGAAACGTAGCCGA |
| mPIR-genotyping F | CCTTGGAGCTGTAGGGAGATAAAT |
| mPIR-genotyping R | GGATCCAAACTCAAGTTGCTAGAC |
| Vill-Cre- genotyping F1 | CGG TTA TTC AAC TTG CAC CA |
| Vill-Cre- genotyping R1 | CCA GTT TCC CTT CTT CCT CTG |
| Vill-Cre- genotyping F2 | AGT GGC CTC TTC CAG AAA TG |
| Vill-Cre- genotyping R2 | TGC GAC TGT GTC TGA TTT CC |
| PLA2G4A CHIP1 F | CAG CTT CAT TTA GTA AGG CG |
| PLA2G4A CHIP1 R | ATA TGT CAT GGT TAG GGT TAA TAT ATA CG |
| PLA2G4A CHIP2 F | GTA TAG ATT CTT TCT TTC ACC AGT TG |
| PLA2G4A CHIP2 R | TAG AGG AGC AGA GAG CAC |
| PLA2G4A CHIP4 F | ATA AGA GTT GAA TGT TGG GCT |
| PLA2G4A CHIP4 R | CAA AGA ATA TAA TTT CAC TAA ATC TTC TGG |
| PLA2G4A CHIP6 F | TTC TGA CTT CAA ACT CCT GG |
| PLA2G4A CHIP6 R | GGA GAA CGC TCT CTT CTC ATA |
| shCtrl | GATGTTGTCAACGACTAGTTT |
| shPirin1 | CCTCCAAGAAAGTTACTCTCT |
| shPirin2 | GCCATTAAGAGAACCAGTTAT |
| hHMOX1 CHIP F (NRF2) | CCCTGCTGAGTAATCCTTTCCCGA |
| hHMOX1 CHIP R (NRF2) | ATGTCCCGACTCCAGACTCCA |
| hNQO1 CHIP F (NRF2) | CCCTTTTAGCCTTGGCACGAAA |
| hNQO1 CHIP R (NRF2) | TGCACCCAGGGAAGTGTGTTGTAT |
| hPIR CHIP F (NRF2) | TTT GCA AAC TGA CCG CCA GCA TTC |
| hPIR CHIP R (NRF2) | ACT GGA CCC ACA CTC TCT TAA CCT |
| hGAPDH CHIP F (Pol II) | GCACGTAGCTCAGGCCTCAAGAC |
| hGAPDH CHIP R (Pol II) | GACTGTCGAACAGGAGGAGCAGAG |
| Plv-EF1a-blast-EcoRI-PIR-Flag hifi-F | CTCTAGATCGCGAACGCGTGATGGGGTCCTCCAAGAAAG |
| Plv-EF1a-blast-EcoRI-PIR-Flag hifi-R | TAGAGCGGCCGCCCTCGAGGCTACTTATCGTCGTCATCCTTG |
| hPIR sgRNA1 F | CAC CGC GAA GCG CTG AGT CAC GGT G |
| hPIR sgRNA1 R | AAA CCA CCG TGA CTC AGC GCT TCG C |
| hPIR sgRNA2 F | CAC CGA CTA GGT GAG GGA ATG CTG G |
| hPIR sgRNA2 R | AAA CCC AGC ATT CCC TCA CCT AGT C |
| hPLA2G4A sgRNA1 F | CACCG ATCTTAGAACTGCATCCAAG |
| hPLA2G4A sgRNA1 R | AAAC CTTGGATGCAGTTCTAAGATC |
| hPLA2G4A sgRNA2 F | CACCG CTTACTATAATGTGCTGGTA |
| hPLA2G4A sgRNA2 R | AAAC TACCAGCACATTATAGTAAGC |
| mPIR sgRNA1 F | CACCGAGGGTGTGTCCCCCGGTCCA |
| mPIR sgRNA1 R | AAACTGGACCGGGGGACACACCCTC |
| mPIR sgRNA2 F | CACCGAGTCTAGGCACCTCCGACTG |
| mPIR sgRNA2 R | AAACCAGTCGGAGGTGCCTAGACTC |
| mPIR sgRNA3 F | CACCGCGTGACTCAGCGCTTTGTTG |
| mPIR sgRNA3 R | AAACCAACAAAGCGCTGAGTCACGC |
| mPIR sgRNA4 F | CACCGCTATCATCTCAAATCGTGG |
| mPIR sgRNA4 R | AAACCCACGATTTGAGATGATAGC |
| pLV-EF1a-PLA2G4A-Flag F | CTCTAGATCGCGAACGCGTGATGTCATTTATAGATCCTTACC |
| pLV-EF1a-PLA2G4A-Flag R | TAGAGCGGCCGCCCTCGAGGCTATGCTTTGGGTTTACTTAG |
| shPLA2G4A1 | CCGACTTATTTGGAAGCAAAT |
| shPLA2G4A2 | CCTCCGTTCAAGGAACTTCTA |
| shPLA2G4A3 | CCTTGTATTCTCACCCTGATT |
